# Supplementary material for: N-Hydroxy-N-Propargylamide Derivatives of Ferulic Acid: Inhibitors of Cholinesterases and Monoamine Oxidases
Source: Molecules. 2022 Nov 1;27(21):7437. doi: 10.3390/molecules27217437 (PMC9655428; doi:10.3390/molecules27217437)
Supplement: Supplementary file 1 [file molecules-27-07437-s001.zip › molecules-1980463-supplementary.pdf]

Supplementary Material

CONTENT

1. NMR, IR and HRMS spectra of compounds 1–6, 15 and 16…………S2–S38

1. NMR, IR and HRMS spectra of compounds 1–6, 15 and 16

Compound 1

^{1}H NMR

^{13}C NMR

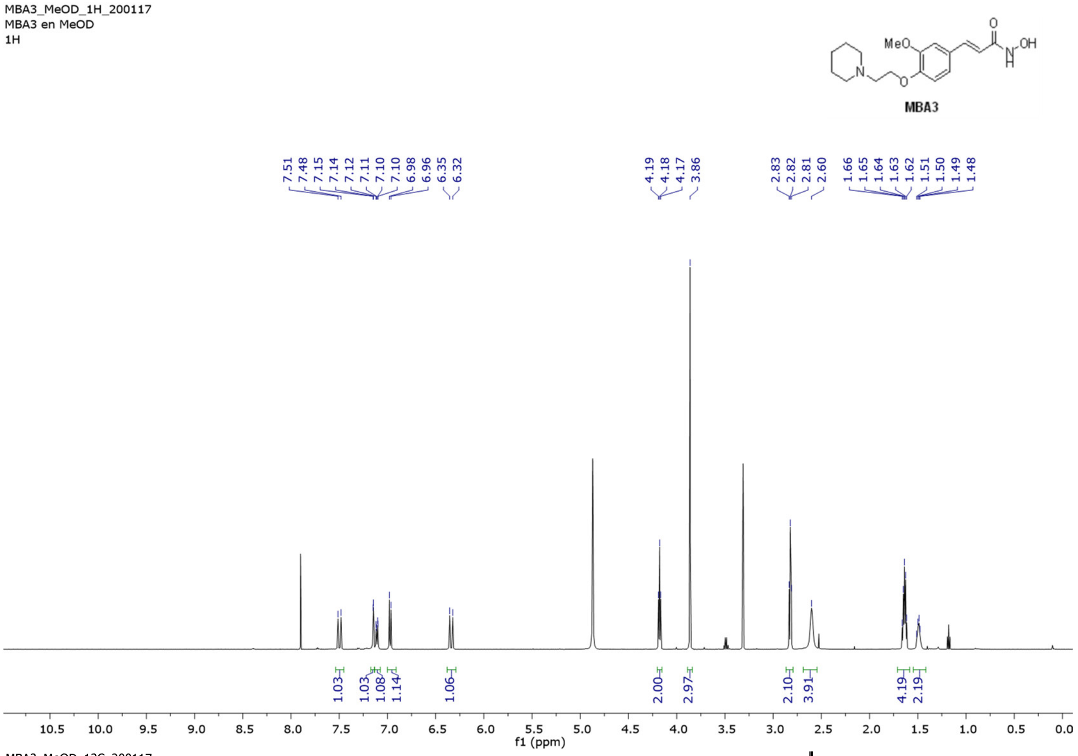

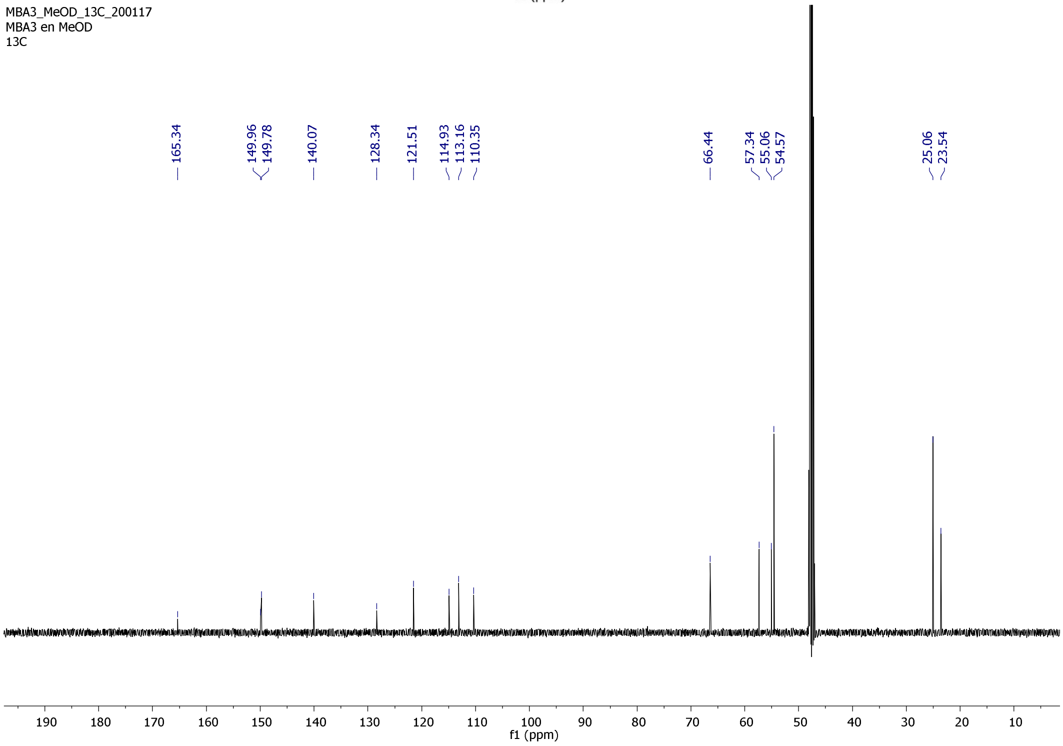

2D COSY NMR

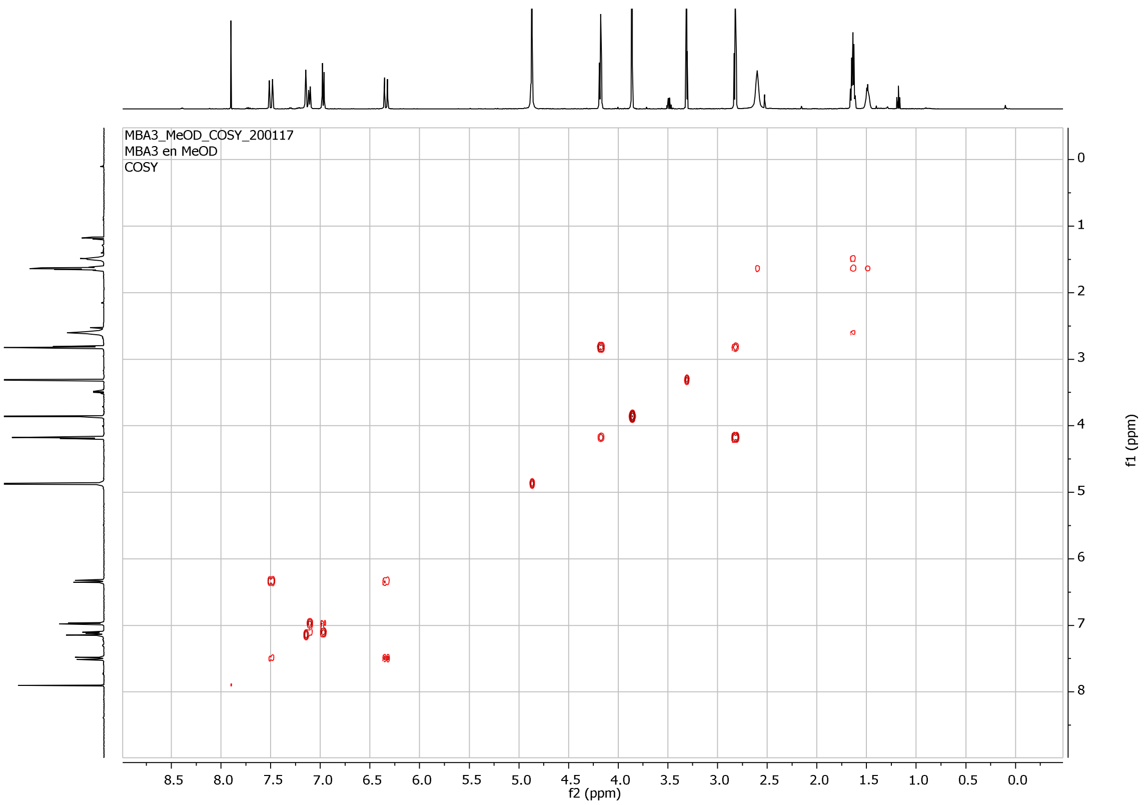

2D HSQC NMR

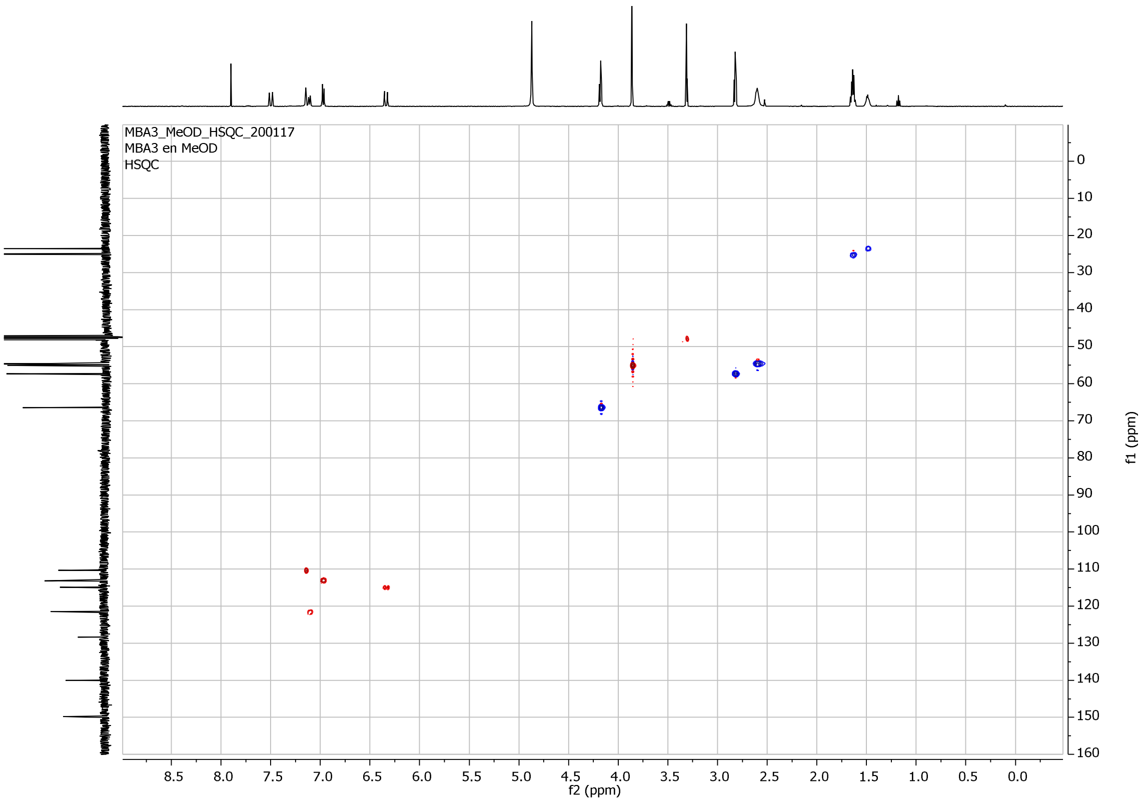

2D HMBC NMR

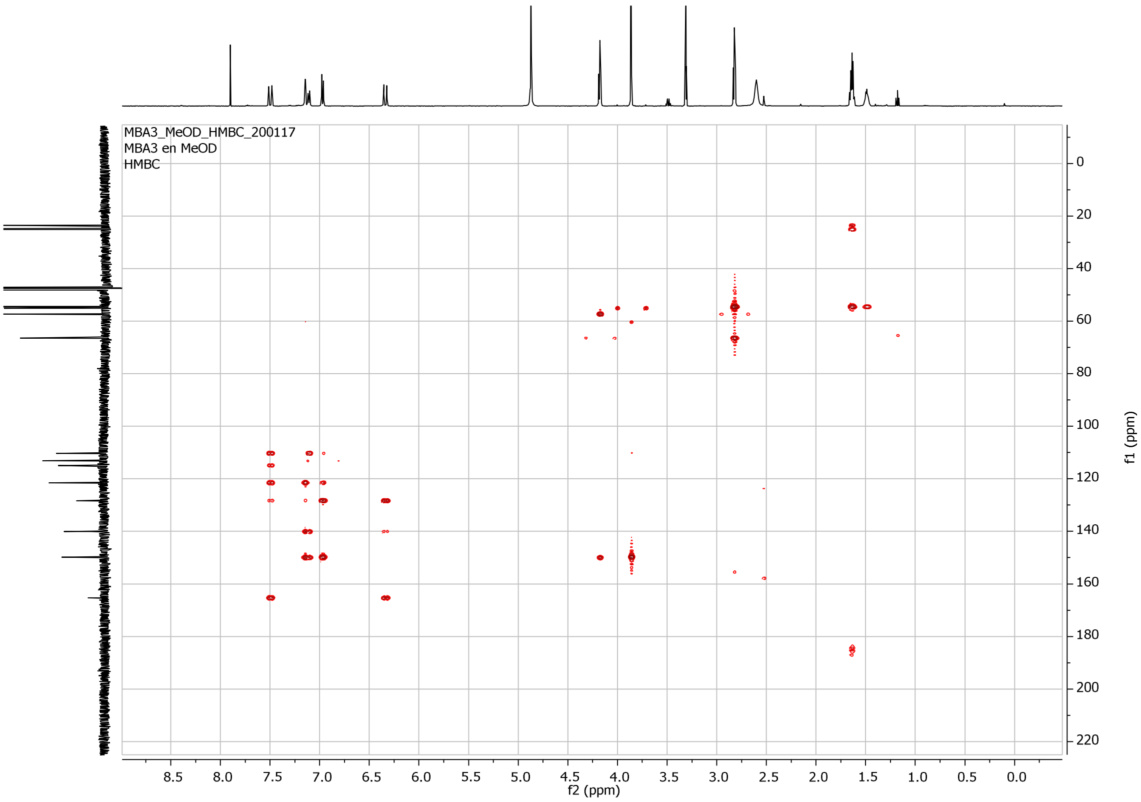

MBA3_MeOD_HMBC_200117
MBA3 en MeOD
HMBC
f2 (ppm)
f1 (ppm)

FTIR

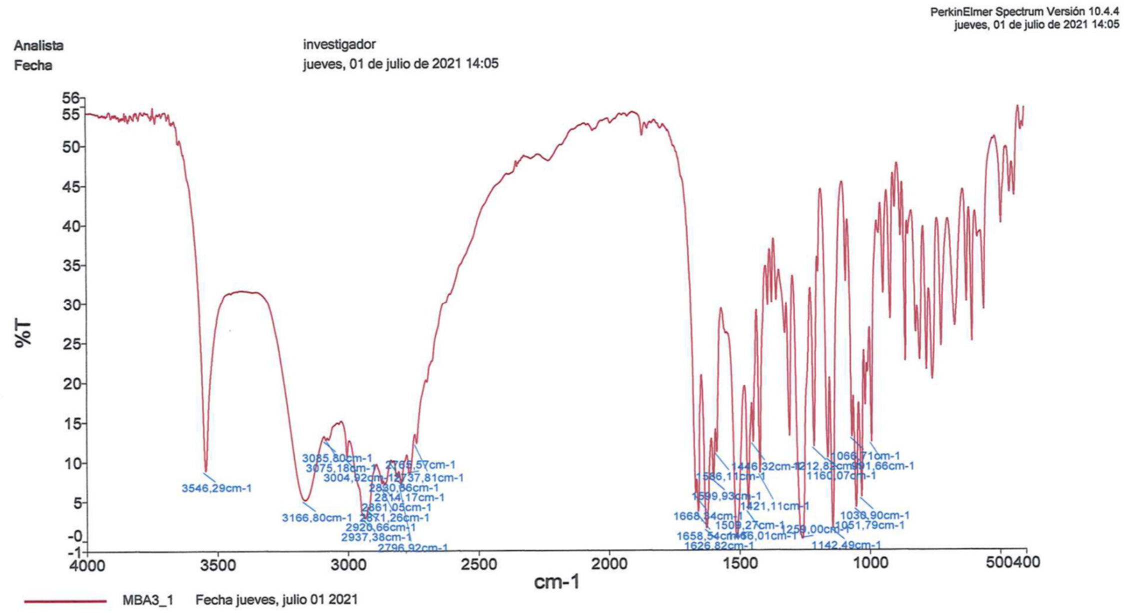

PerkinElmer Spectrum Versión 10.4.4
jueves, 01 de julio de 2021 14:05
Analista
Fecha
investigador
jueves, 01 de julio de 2021 14:05
%T
cm-1
3546,29cm-1
3166,80cm-1
MBA3_1
Fecha jueves, julio 01 2021

HRMS

Qualitative Compound Report

**Data File**	247_MBA3_02.d	**Sample Name**	MBA3
**Sample Type**	Sample	**Position**	Vial 1
**Instrument Name**	Instrument 1	**User Name**	
**Acq Method**	ESI_ACN_75_pos_new.m	**Acquired Time**	2/19/2021 1:23:41 PM (UTC+01:00)
**IRM Calibration Status**	Success	**DA Method**	Defecto_modificado.m
**Comment**			

**Sample Group**		**Info.**	
**User**	DANIEL DIEZ	**Stream Name**	LC 1
**Acquisition Time (Local)**	2/19/2021 1:23:41 PM (UTC+01:00)	**Acquisition SW Version**	6200 series TOF/6500 series Q-TOF B.08.00 (B8058.3 SP1)
**QTOF Driver Version**	8.00.00	**QTOF Firmware Version**	2.712
**Tune Mass Range Max.**	1700		

Compound Table

Compound Label	RT	Mass	Abund	Formula	Tgt Mass	Diff (ppm)	Hits (DB)
Cpd 1: C17 H24 N2 O4; 0.837	0.837	320.1739	15628177	C17 H24 N2 O4	320.1736	0.84	1

Compound Label	*m/z*	RT	Algorithm	Mass
Cpd 1: C17 H24 N2 O4; 0.837	321.181	0.837	Find by Formula	320.1739

MS Zoomed Spectrum

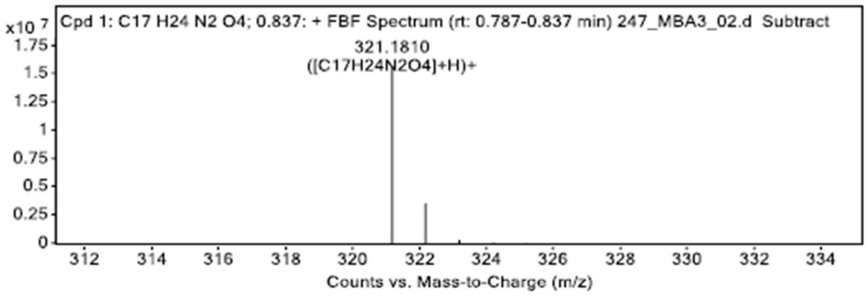

MS Spectrum Peak List

m/z	z	Abund	Formula	Ion
321.181	1	15628177	C17H24N2O4	(M+H)+
322.1839	1	3490927	C17H24N2O4	(M+H)+
323.1983	1	242480.19	C17H24N2O4	(M+H)+
324.2019	1	29219.22	C17H24N2O4	(M+H)+
325.1893	1	1536.25	C17H24N2O4	(M+H)+

MS Zoomed Spectrum

Qualitative Compound Report

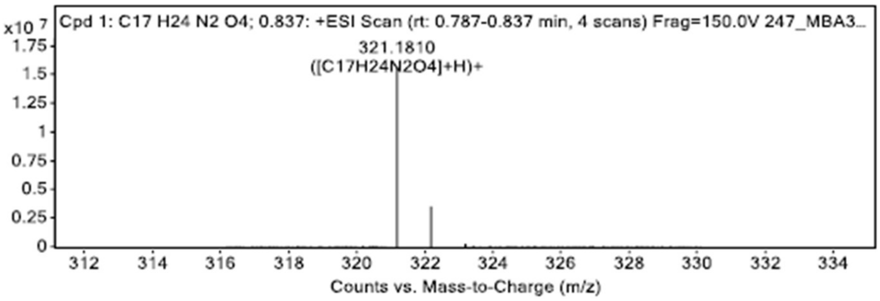

MS Spectrum Peak List

m/z	*Calc m/z*	Diff(ppm)	z	Abund	Formula	Ion
321.181	321.1809	0.48	1	15628177	C17H24N2O4	(M+H)+
322.1839	322.1841	-0.4	1	3490927	C17H24N2O4	(M+H)+
323.1983	323.1866	36.36	1	242480.19	C17H24N2O4	(M+H)+
324.2019	324.1891	39.48	1	29219.22	C17H24N2O4	(M+H)+
325.1893	325.1916	-7.26	1	1536.25	C17H24N2O4	(M+H)+

--- End Of Report ---

Compound 2

^{1}H NMR

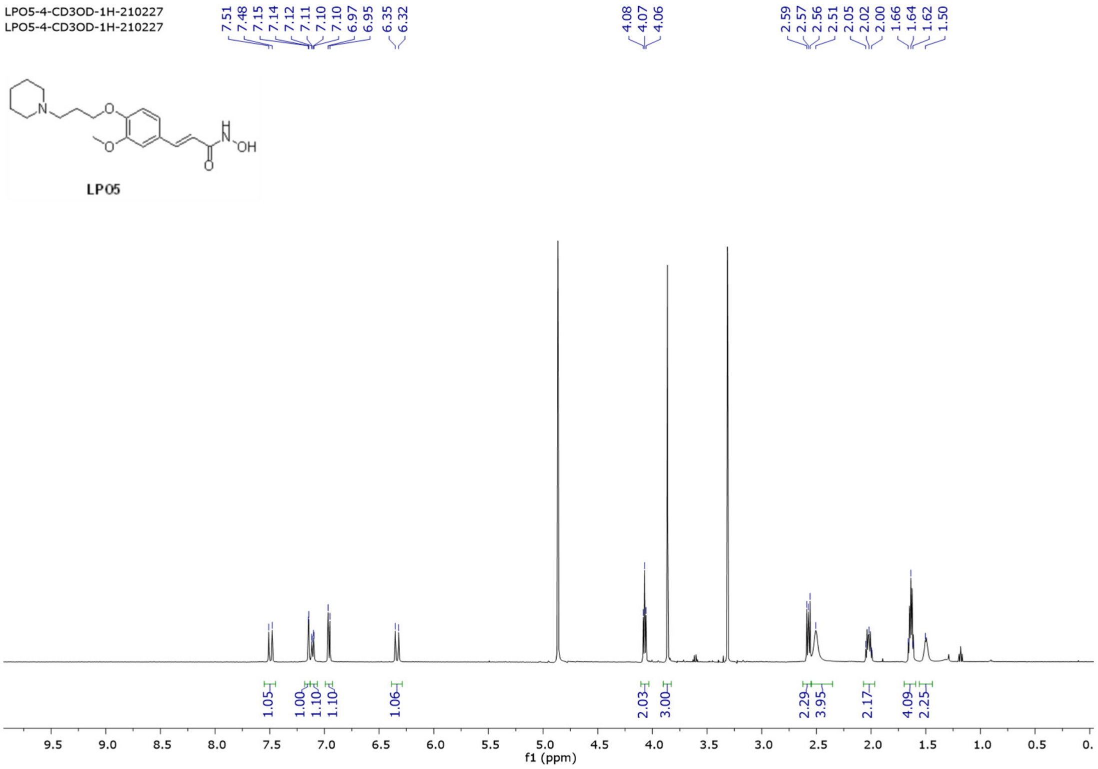

^{13}C NMR

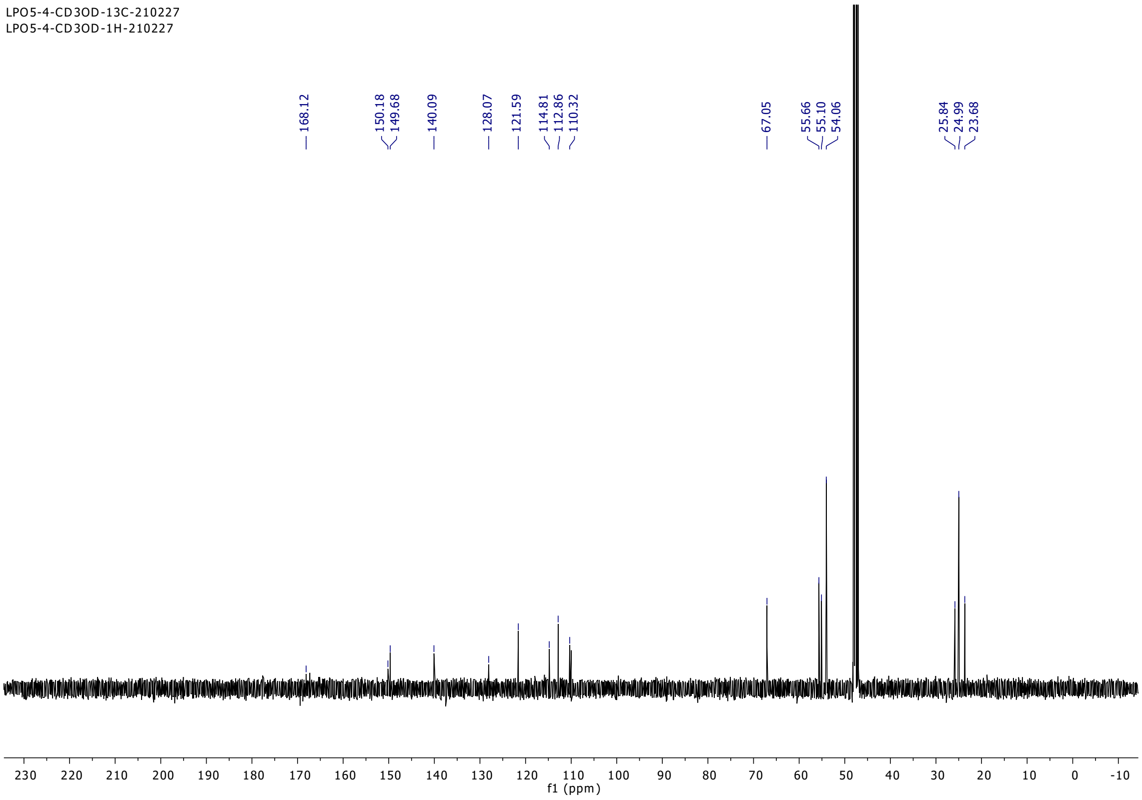

2D COSY NMR

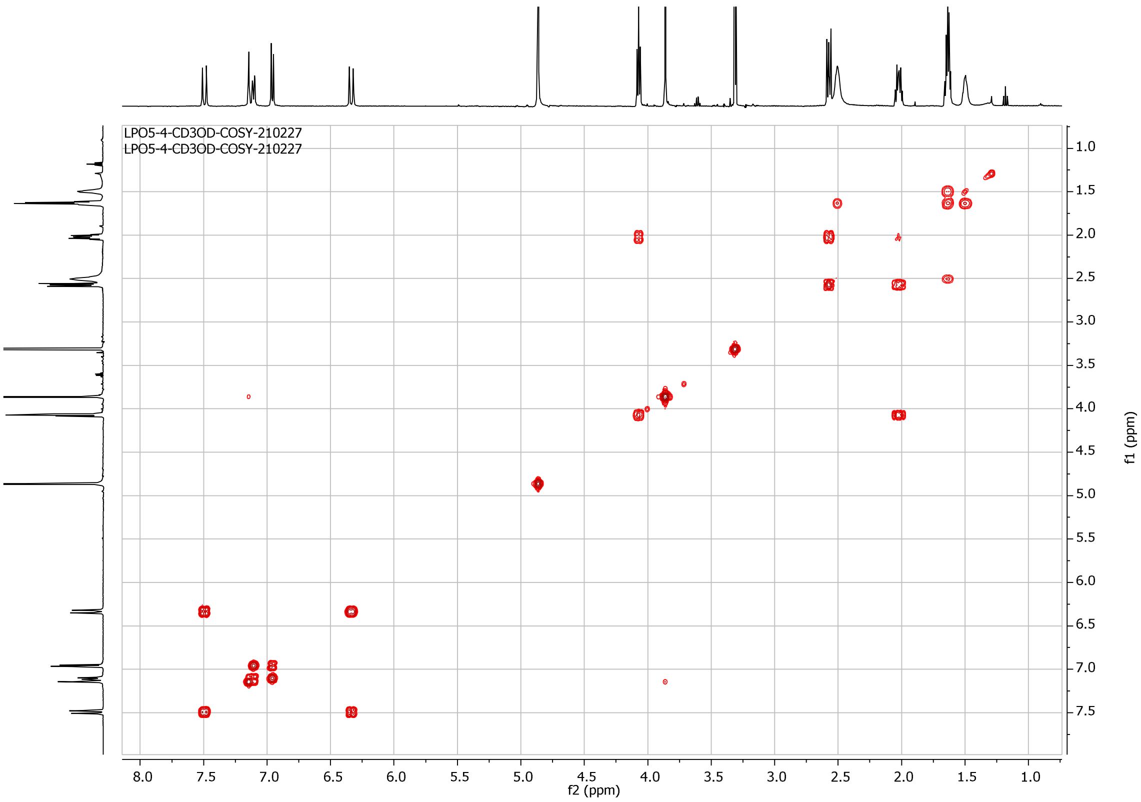

2D HSQC NMR

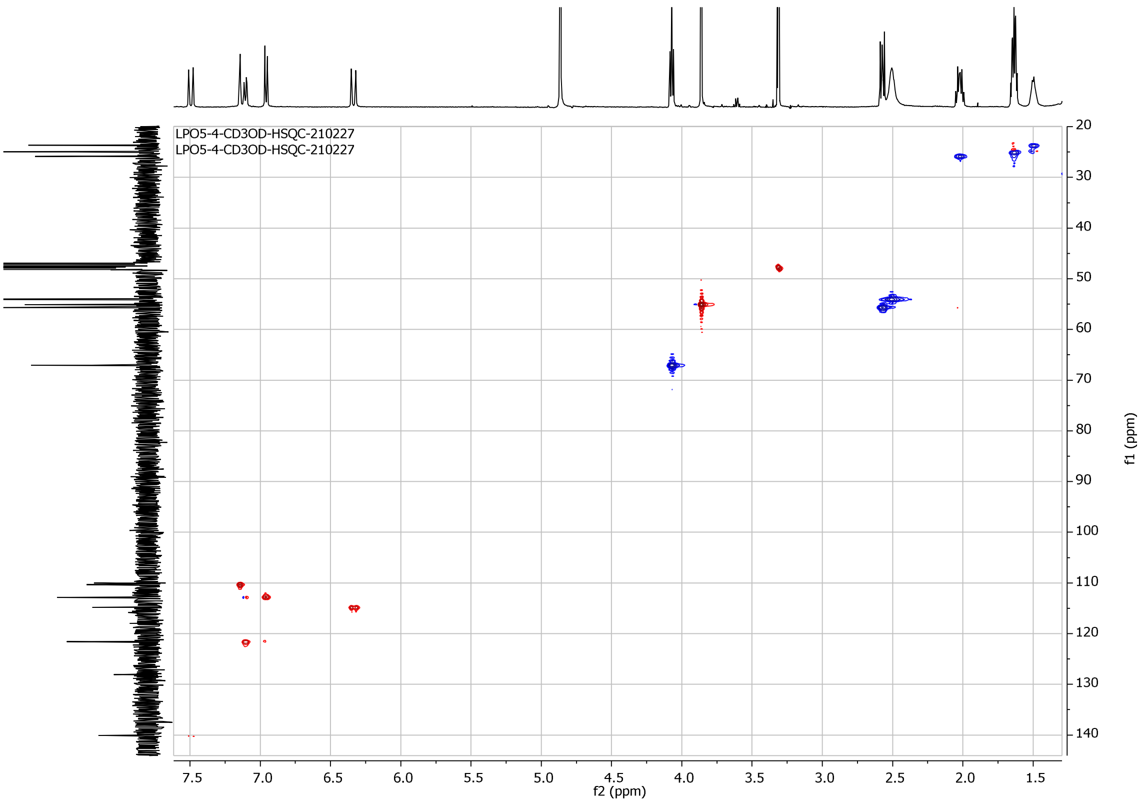

2D HMBC NMR

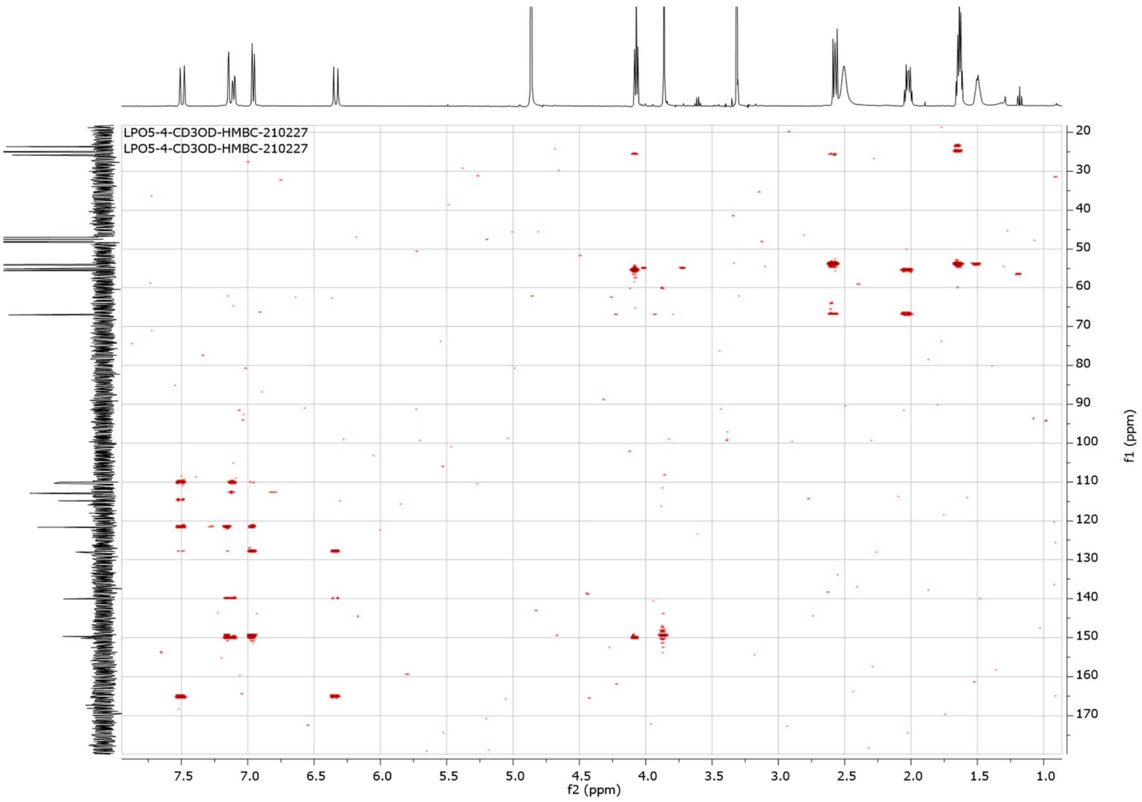

FTIR

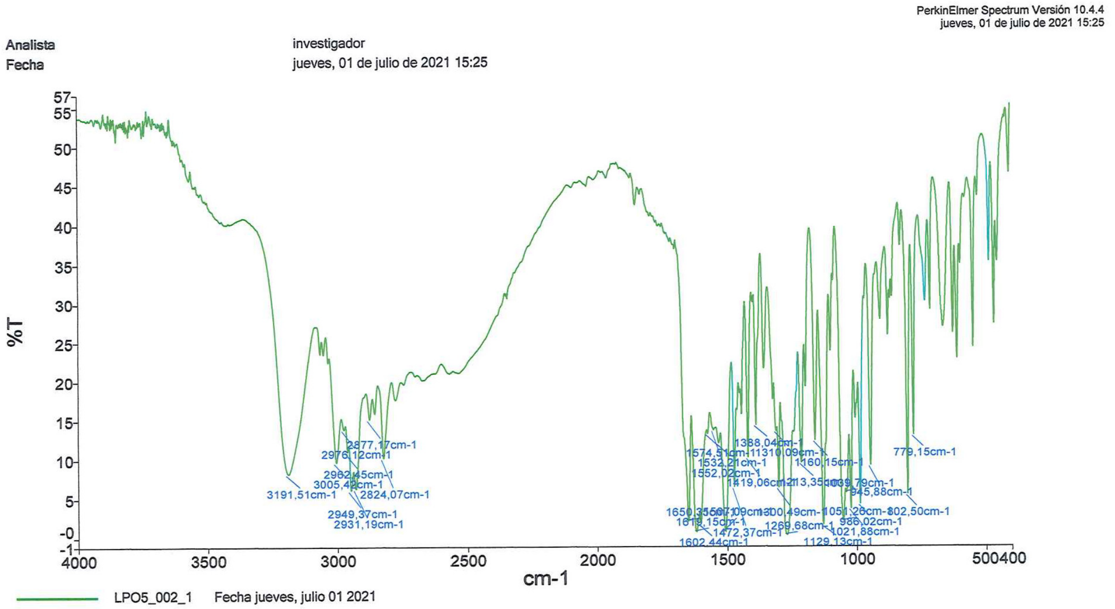

HRMS

Qualitative Compound Report

**Data File**	255_LPO5_01.d	**Sample Name**	LPO5
**Sample Type**	Sample	**Position**	Vial 9
**Instrument Name**	Instrument 1	**User Name**	
**Acq Method**	ESI_ACN_75_pos_new.m	**Acquired Time**	2/19/2021 11:57:57 AM (UTC+01:00)
**IRM Calibration Status**	Success	**DA Method**	Defecto_modificado.m
**Comment**			

**Sample Group**		**Info.**	
**User**	DANIEL DIEZ	**Stream Name**	LC 1
**Acquisition Time (Local)**	2/19/2021 11:57:57 AM (UTC+01:00)	**Acquisition SW Version**	6200 series TOF/6500 series Q-TOF B.08.00 (B8058.3 SP1)
**QTOF Driver Version**	8.00.00	**QTOF Firmware Version**	2.712
**Tune Mass Range Max.**	1700		

Compound Table

Compound Label	RT	Mass	Abund	Formula	Tgt Mass	Diff (ppm)	Hits (DB)
Cpd 1: C18 H26 N2 O4; 2.698	2.698	334.1909	13238040	C18 H26 N2 O4	334.1893	4.92	1

Compound Label	*m/z*	RT	Algorithm	Mass
Cpd 1: C18 H26 N2 O4; 2.698	335.1977	2.698	Find by Formula	334.1909

MS Zoomed Spectrum

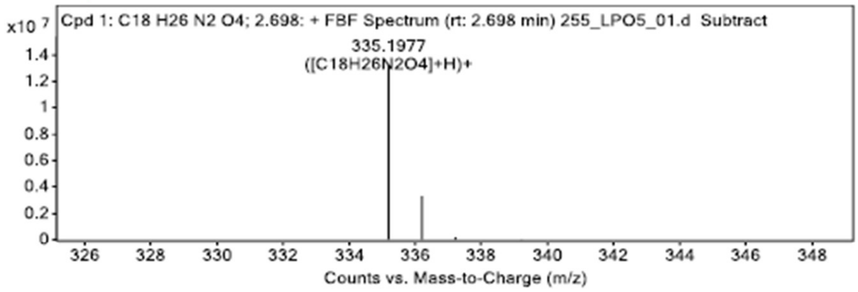

MS Spectrum Peak List

m/z	z	Abund	Formula	Ion
335.1977	1	13238040	C18H26N2O4	(M+H)+
336.2028	1	3285623.5	C18H26N2O4	(M+H)+
337.2164	1	180252.14	C18H26N2O4	(M+H)+
339.2135	1	4773.48	C18H26N2O4	(M+H)+

MS Zoomed Spectrum

Qualitative Compound Report

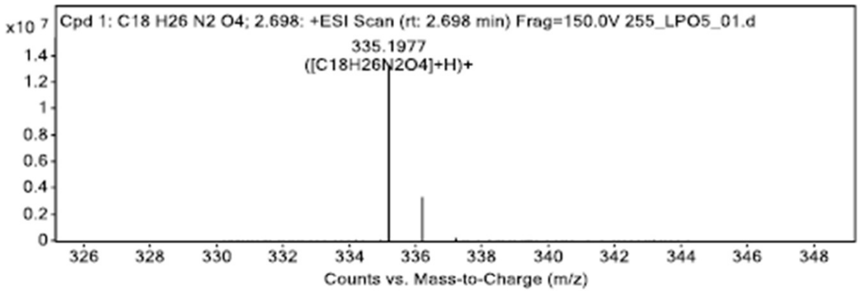

MS Spectrum Peak List

m/z	*Calc m/z*	Diff(ppm)	z	Abund	Formula	Ion
335.1977	335.1965	3.34	1	13238040	C18H26N2O4	(M+H)+
336.2028	336.1997	9.13	1	3285623.5	C18H26N2O4	(M+H)+
337.2164	337.2023	41.73	1	180252.14	C18H26N2O4	(M+H)+
339.2135	339.2074	17.91	1	4773.48	C18H26N2O4	(M+H)+

--- End Of Report ---

Compound 3

^{1}H NMR

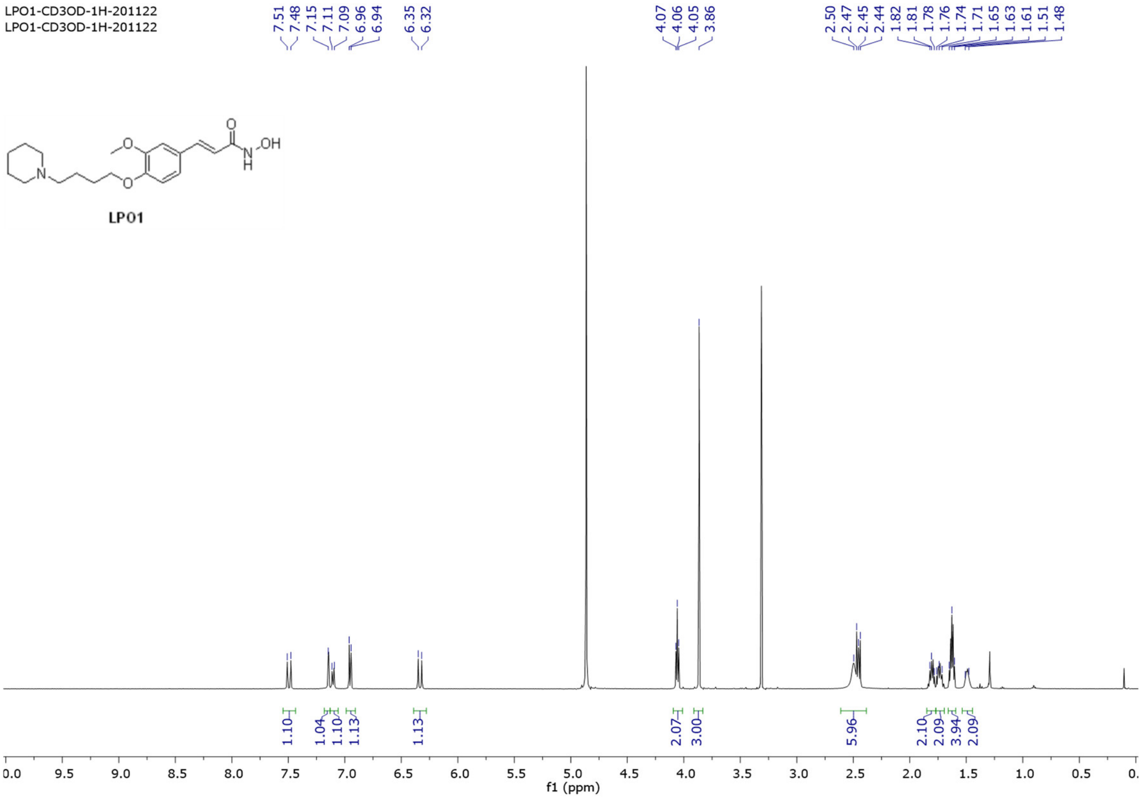

^{13}C NMR

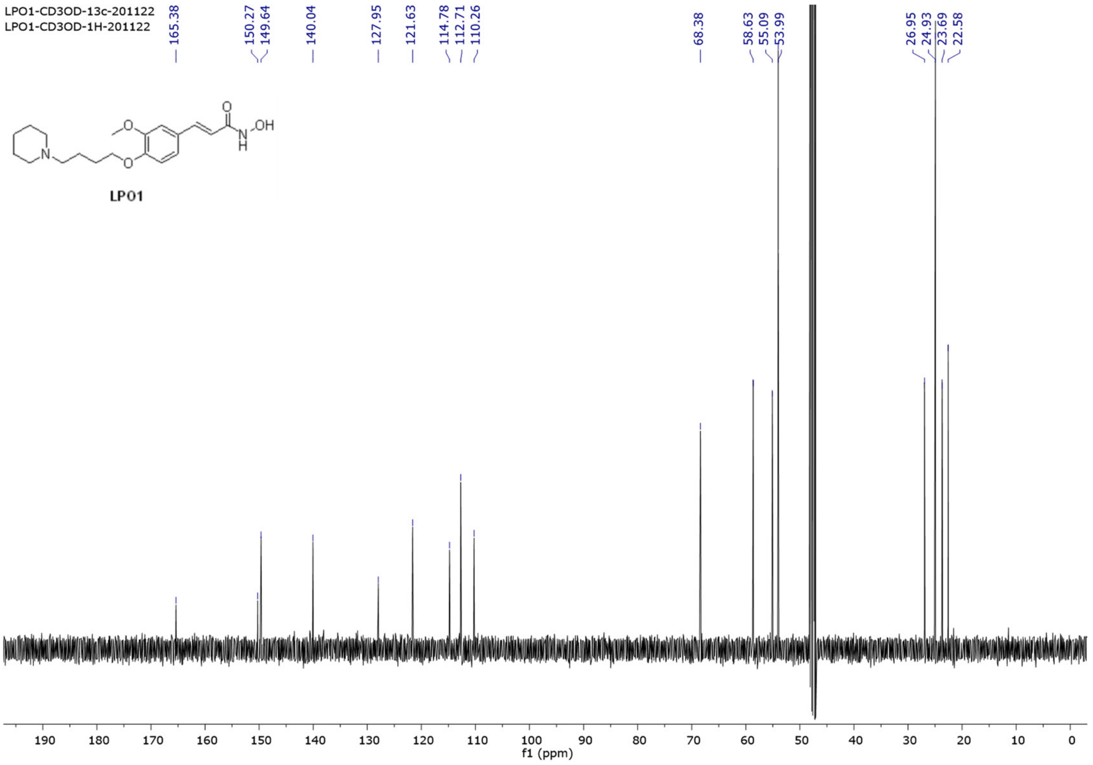

2D COSY NMR

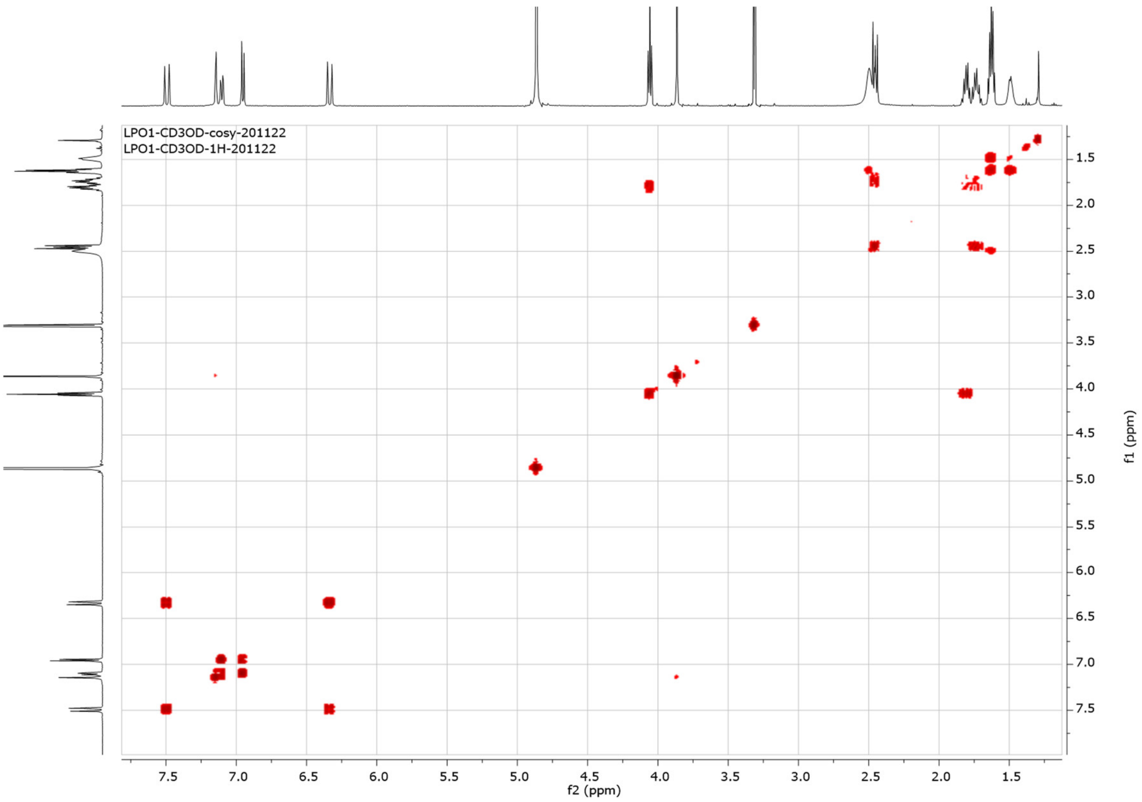

2D HSQC NMR

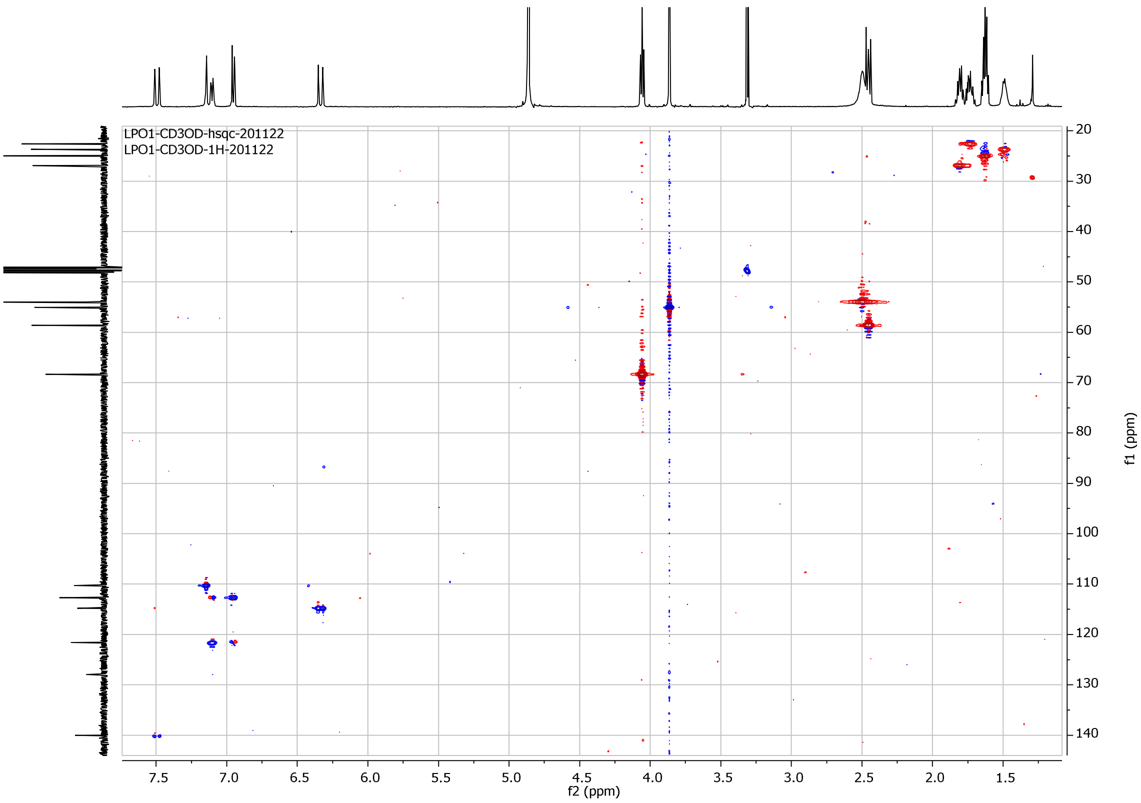

2D HMBC NMR

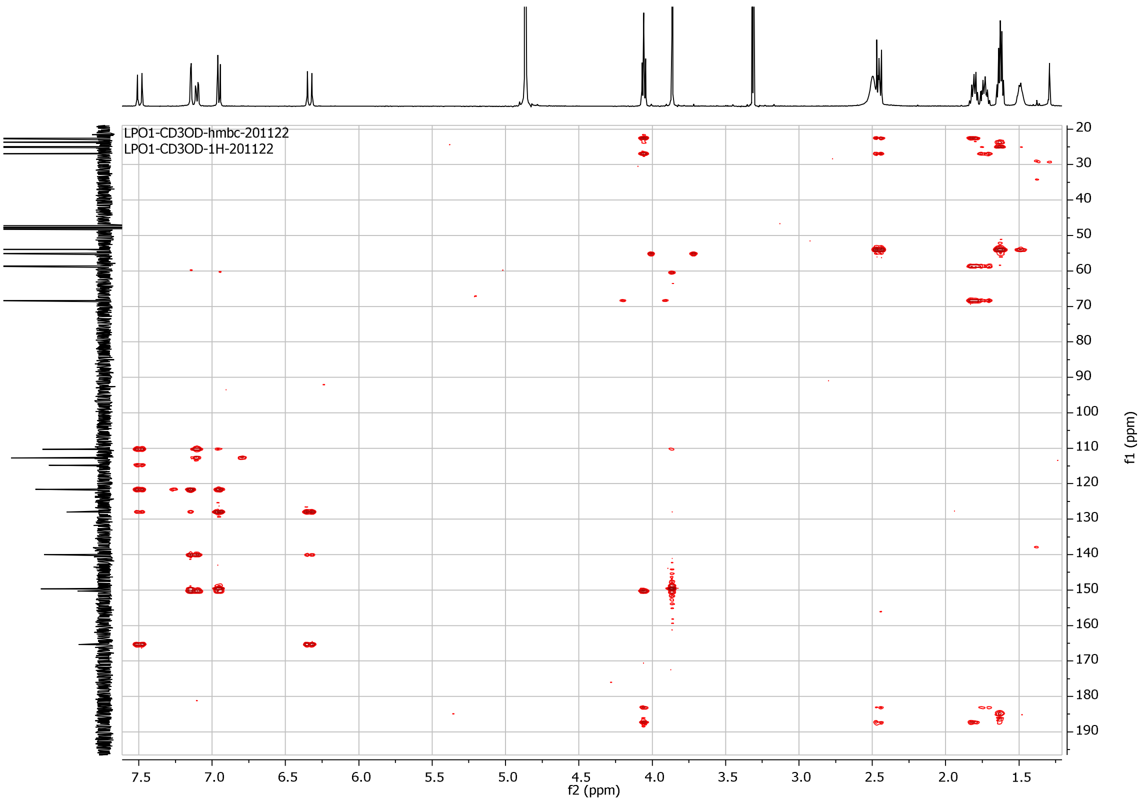

FTIR

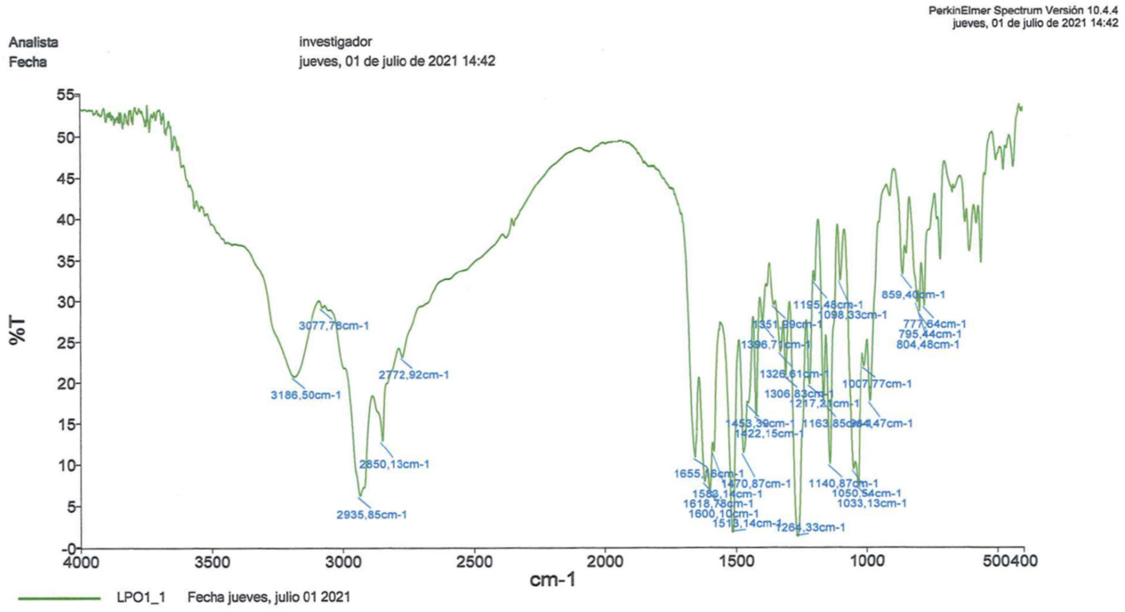

HRMS

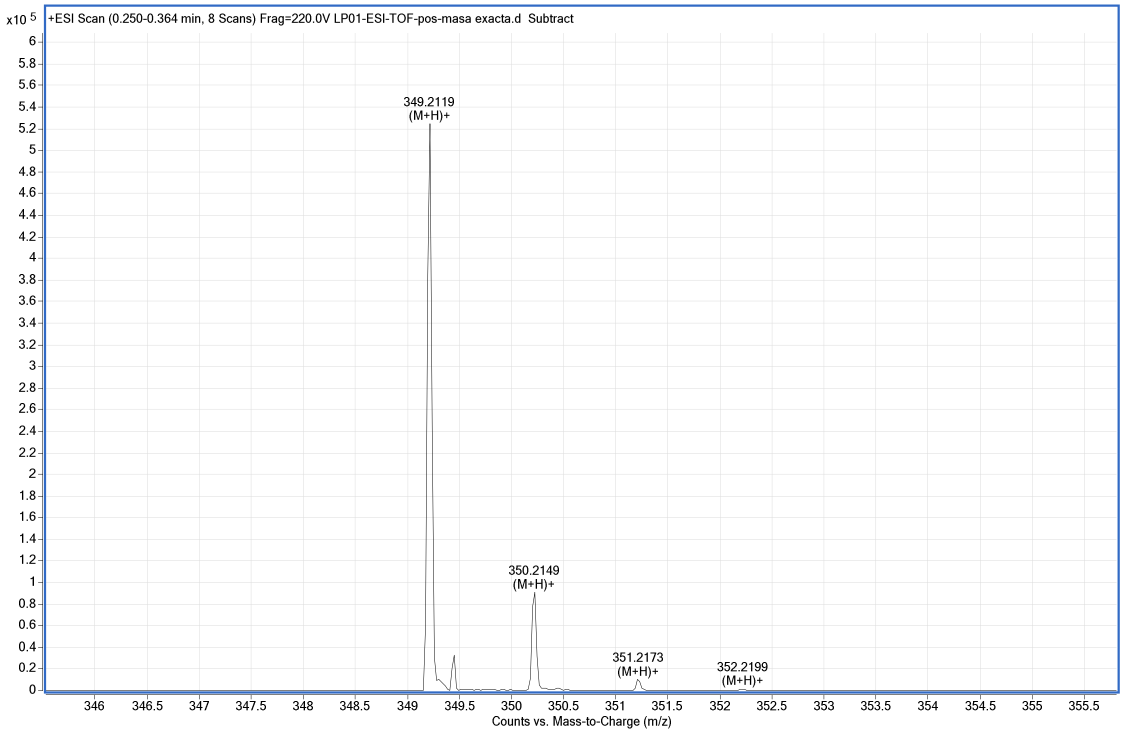

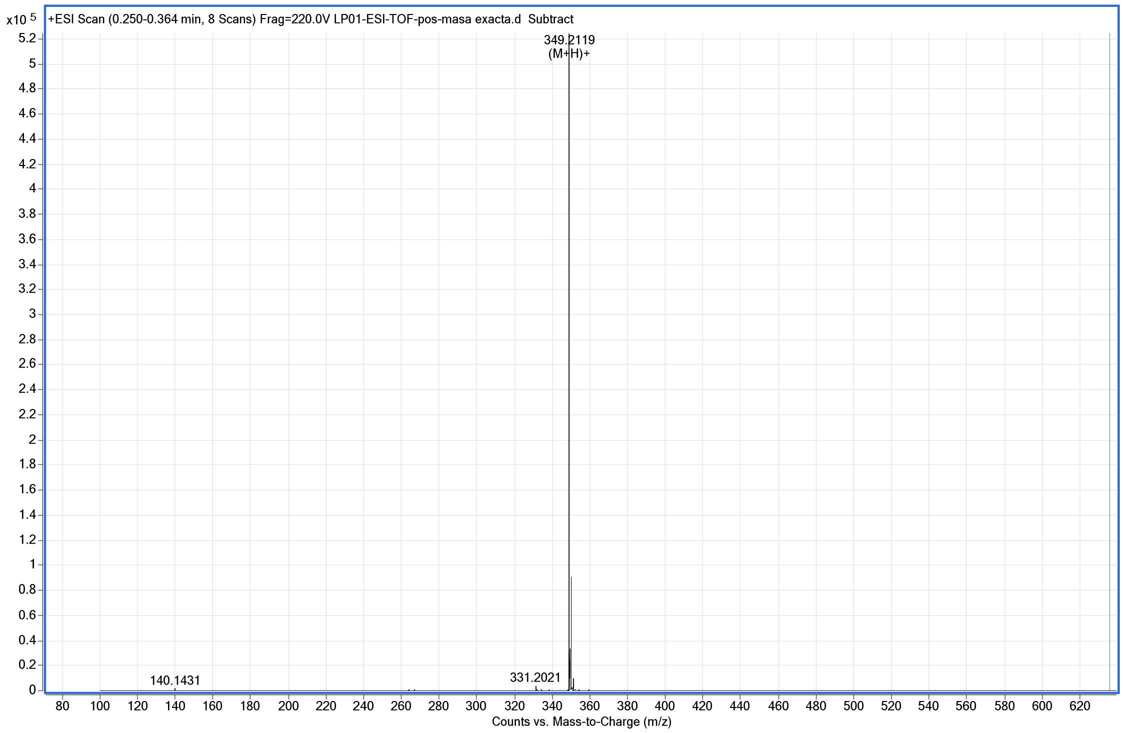

Best	Name	Formula	Score	Mass	Mass (Tgt)	Mass (DB)	Mass (MFG)	Diff (ppm)	Diff (abs. ppm)	Diff (mDa)
		C19 H28 N2 O4	96.17	348.2046			348.2049	0.86	0.86	0.3

Species	Ion Formula	m/z	Height	Score (MFG)	Score (MFG, MS)	Score (MFG, MS/MS)	Score (MFG, mass)	Score (MFG, abund)	Score (MFG, iso. spacing)
(M+H)+	C19 H29 N2 O4	349.2122	524306.3	96.17	96.17		99.43	87.64	99.87

m/z	m/z (Calc)	Diff (ppm)	Diff (mDa)	Height	Height (Calc)	Height %	Height % (Calc)	Height Sum %	Height Sum% (Calc)
349.2119	349.2122	0.86	0.3	530318.7	508024.2	100	100	83.4	79.9
350.2149	350.2154	1.27	0.4	94060.8	110578.8	17.7	21.8	14.8	17.4
351.2173	351.218	2.03	0.7	10364.4	15638.9	2	3.1	1.6	2.5
352.2199	352.2206	1.91	0.7	1158.3	1660.2	0.2	0.3	0.2	0.3

Compound 4

^{1}H NMR

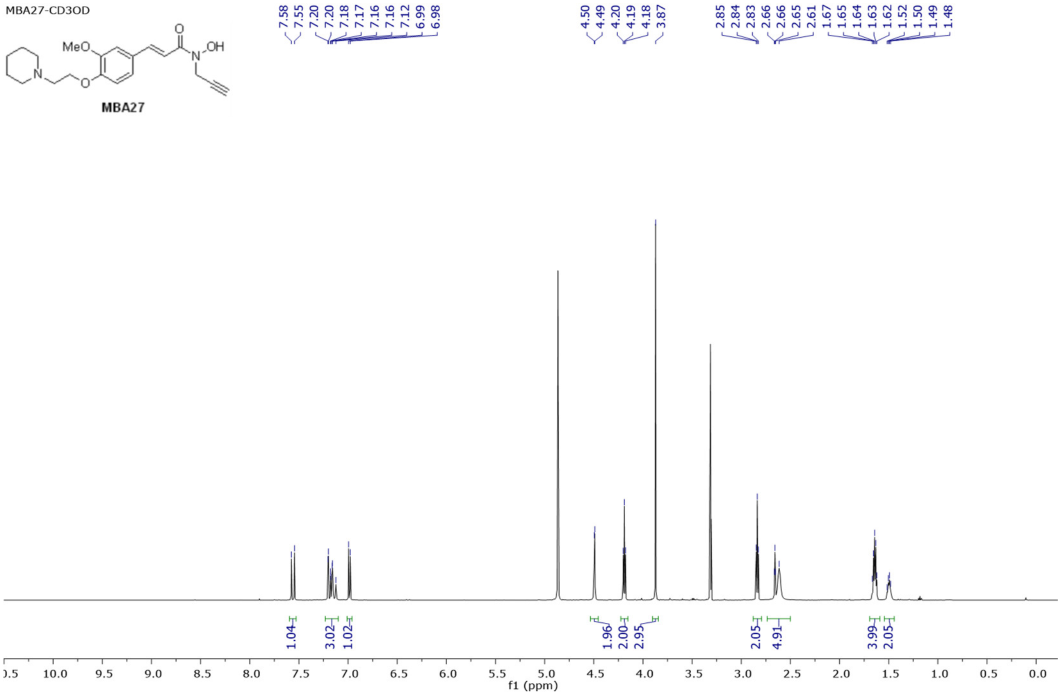

^{13}C NMR

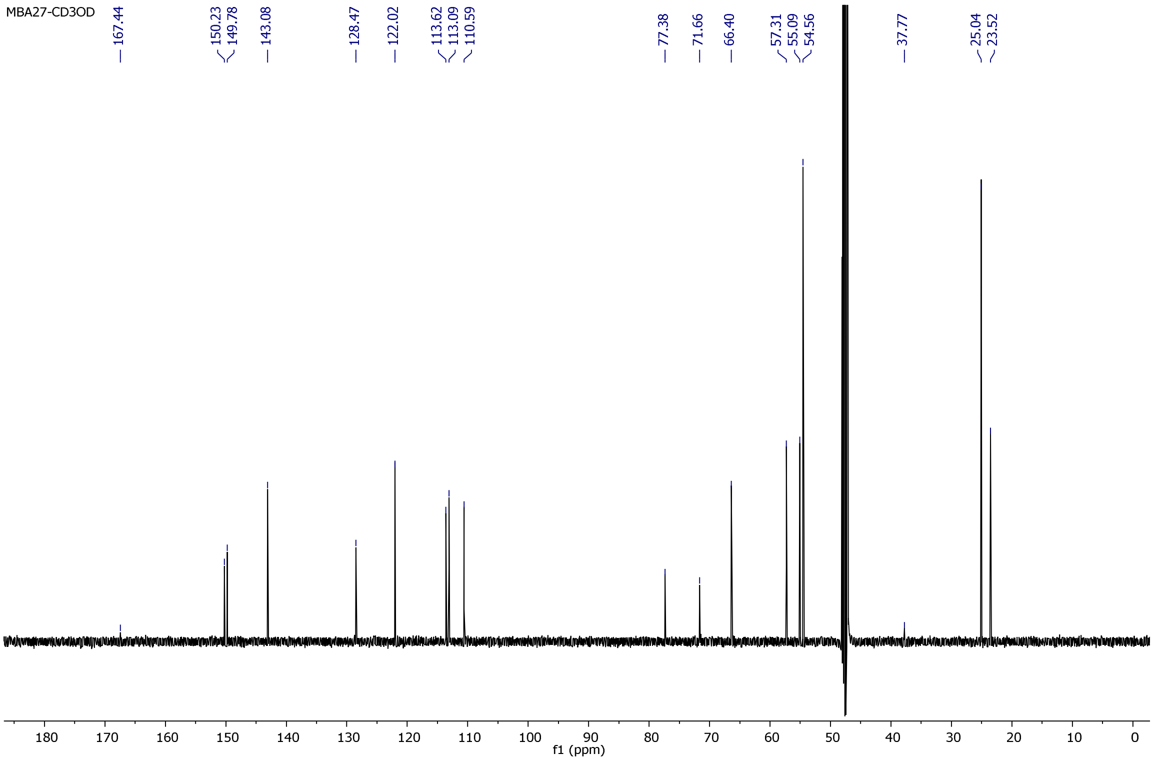

2D COSY NMR

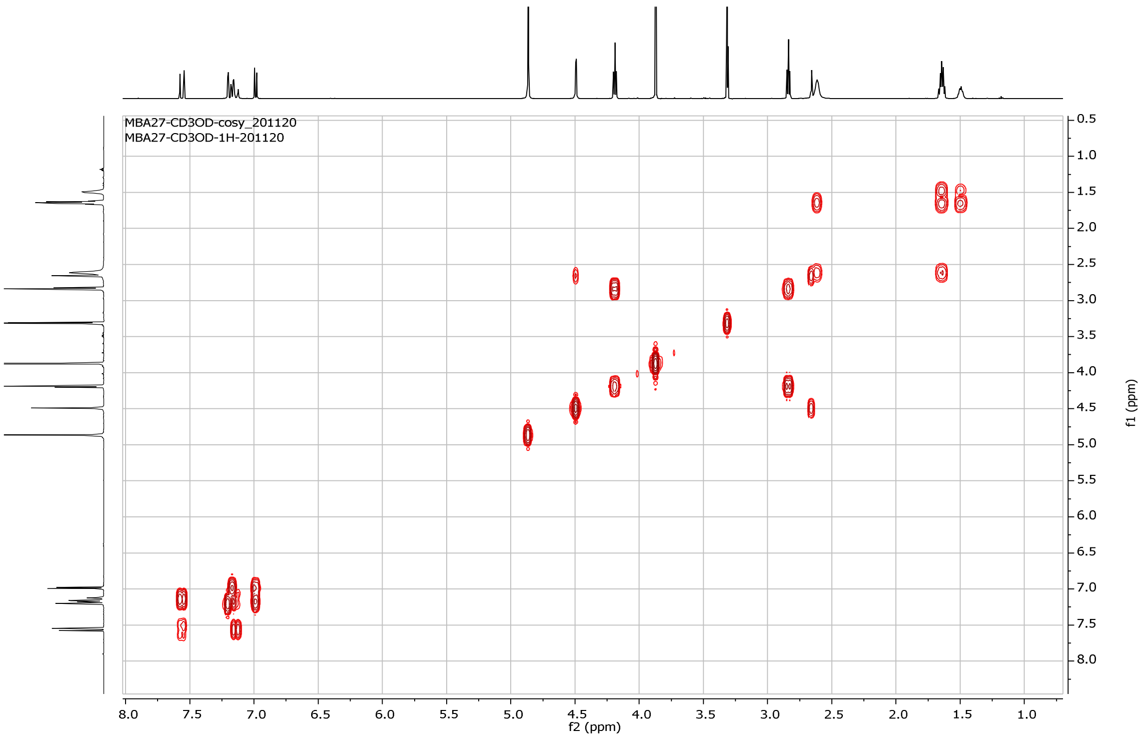

2D HSQC NMR

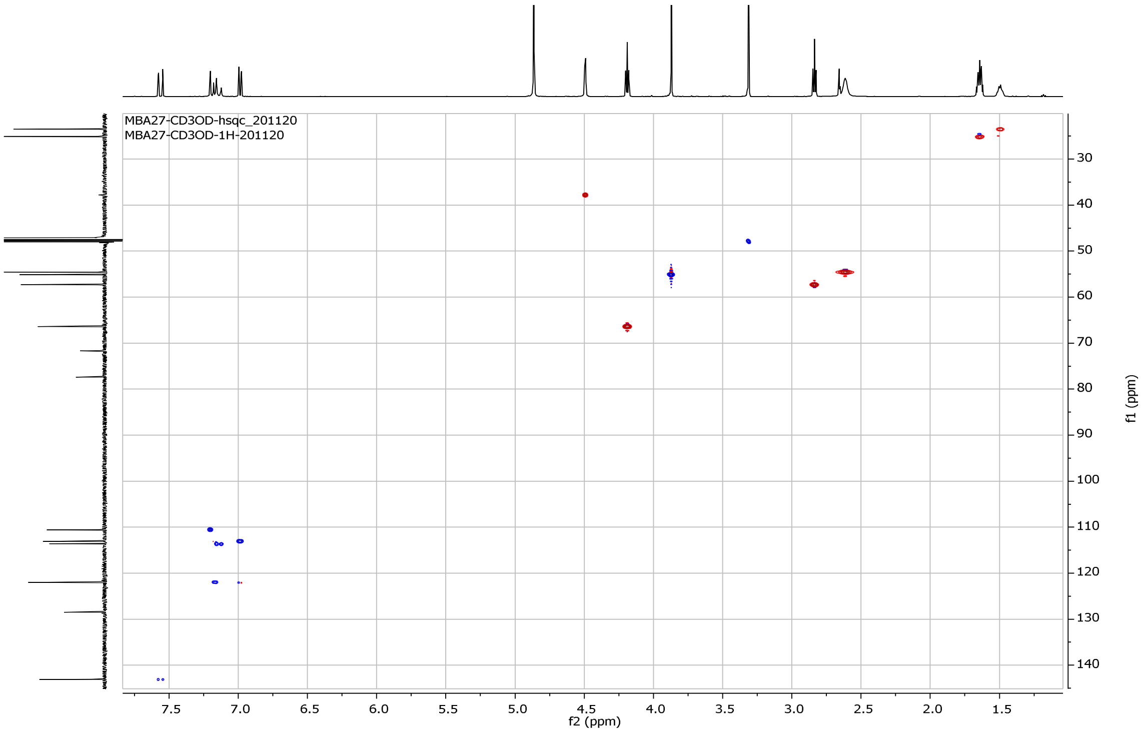

2D HMBC NMR

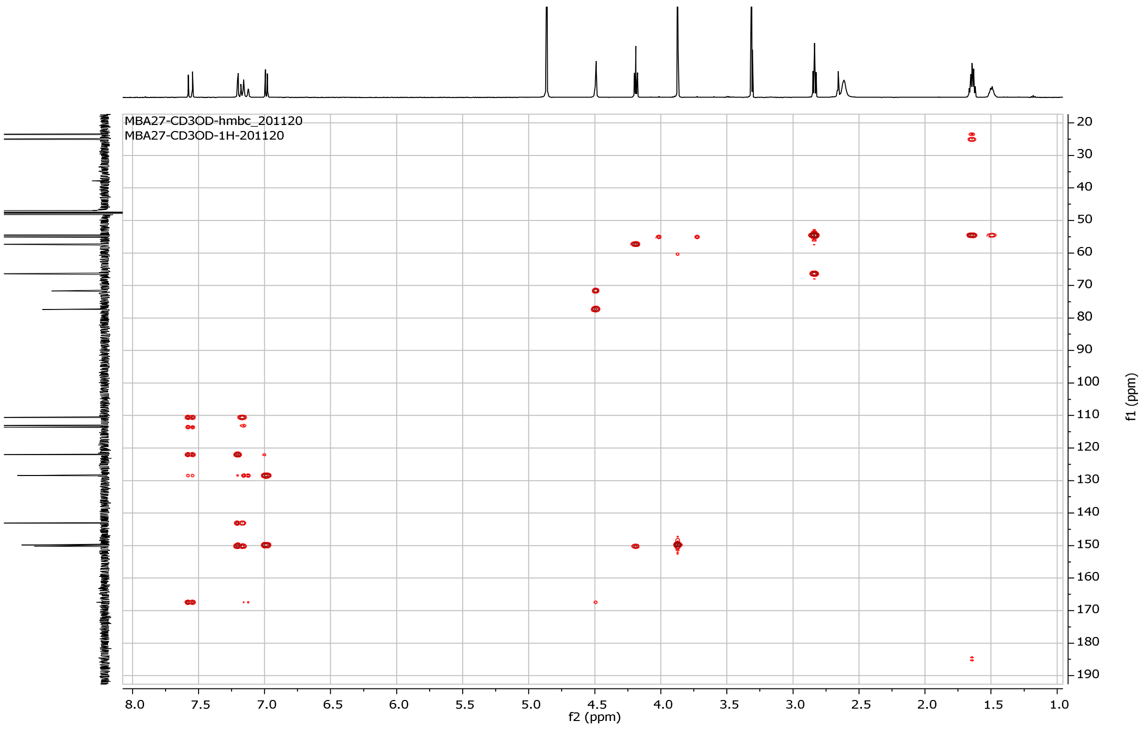

FTIR

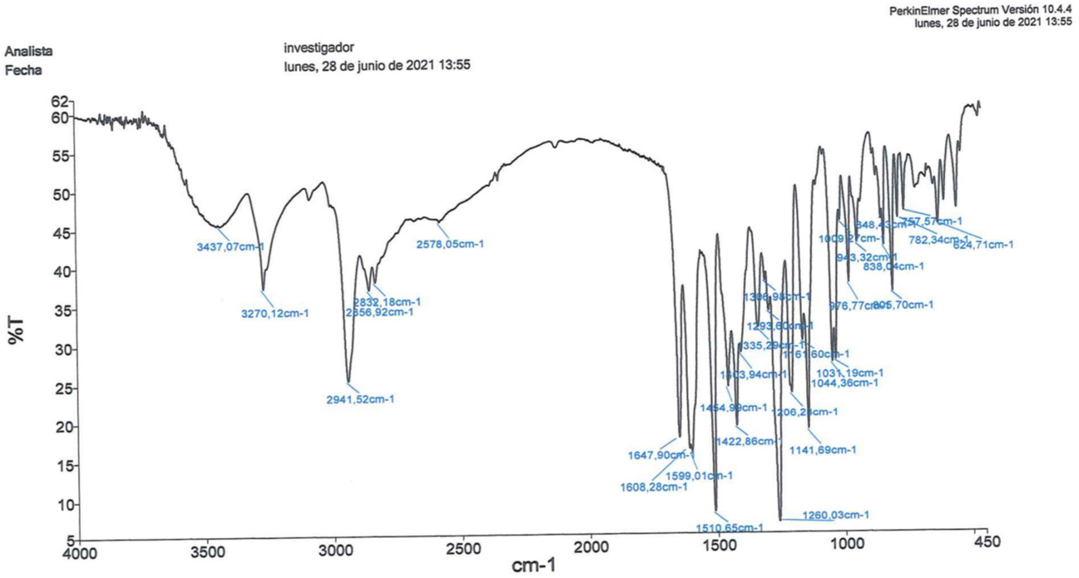

HRMS

Qualitative Compound Report

**Data File**	248_MBA27_01.d	**Sample Name**	MBA27
**Sample Type**	Sample	**Position**	Vial 2
**Instrument Name**	Instrument 1	**User Name**	
**Acq Method**	ESI_ACN_75_pos_new.m	**Acquired Time**	2/19/2021 11:07:10 AM (UTC+01:00)
**IRM Calibration Status**	Success	**DA Method**	Defecto_modificado.m
**Comment**			

**Sample Group**		**Info.**	
**User**	DANIEL DIEZ	**Stream Name**	LC 1
**Acquisition Time (Local)**	2/19/2021 11:07:10 AM (UTC+01:00)	**Acquisition SW Version**	6200 series TOF/6500 series Q-TOF B.08.00 (B8058.3 SP1)
**QTOF Driver Version**	8.00.00	**QTOF Firmware Version**	2.712
**Tune Mass Range Max.**	1700		

Compound Table

Compound Label	RT	Mass	Abund	Formula	Tgt Mass	Diff (ppm)	Hits (DB)
Cpd 1: C20 H26 N2 O4; 0.418	0.418	358.1905	10284418	C20 H26 N2 O4	358.1893	3.38	1

Compound Label	*m/z*	RT	Algorithm	Mass
Cpd 1: C20 H26 N2 O4; 0.418	359.1975	0.418	Find by Formula	358.1905

MS Zoomed Spectrum

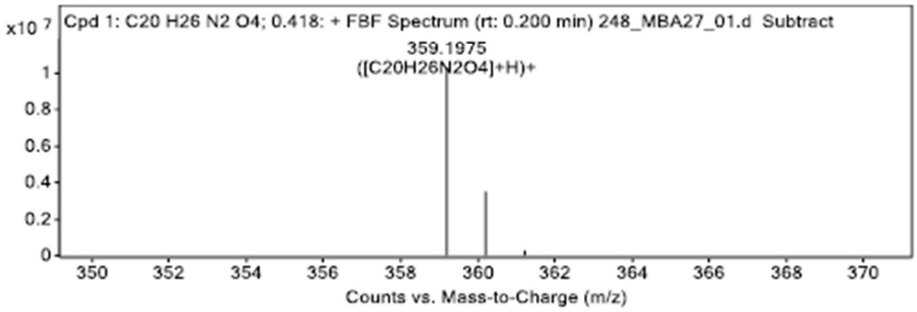

MS Spectrum Peak List

m/z	z	Abund	Formula	Ion
359.1975	1	10284418	C20H26N2O4	(M+H)+
360.2004	1	3511802.75	C20H26N2O4	(M+H)+
361.2185	1	278680.59	C20H26N2O4	(M+H)+

MS Zoomed Spectrum

Qualitative Compound Report

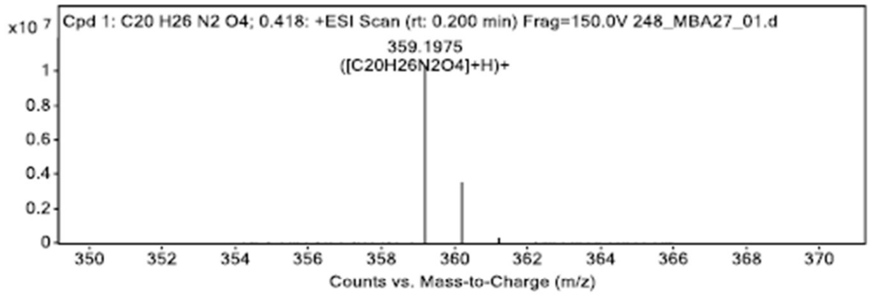

MS Spectrum Peak List

m/z	*Calc m/z*	Diff(ppm)	z	Abund	Formula	Ion
359.1975	359.1965	2.73	1	10284418	C20H26N2O4	(M+H)+
360.2004	360.1997	1.97	1	3511802.75	C20H26N2O4	(M+H)+
361.2185	361.2024	44.47	1	278680.59	C20H26N2O4	(M+H)+

--- End Of Report ---

Compound 5

¹H NMR

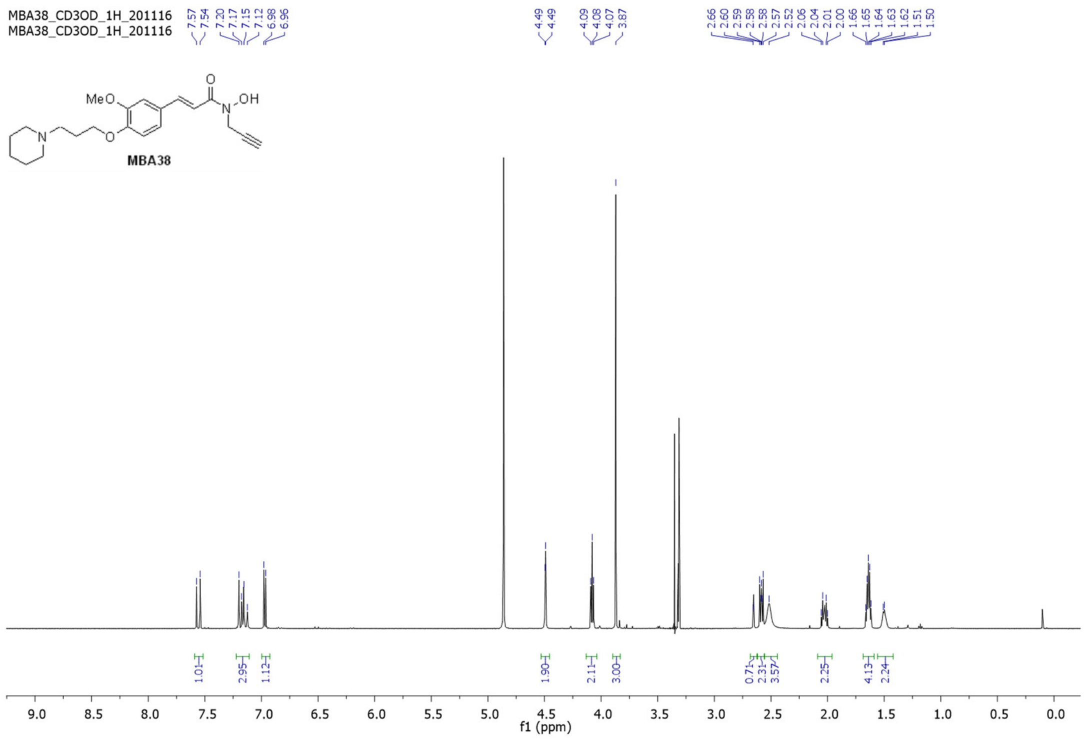

¹³C NMR

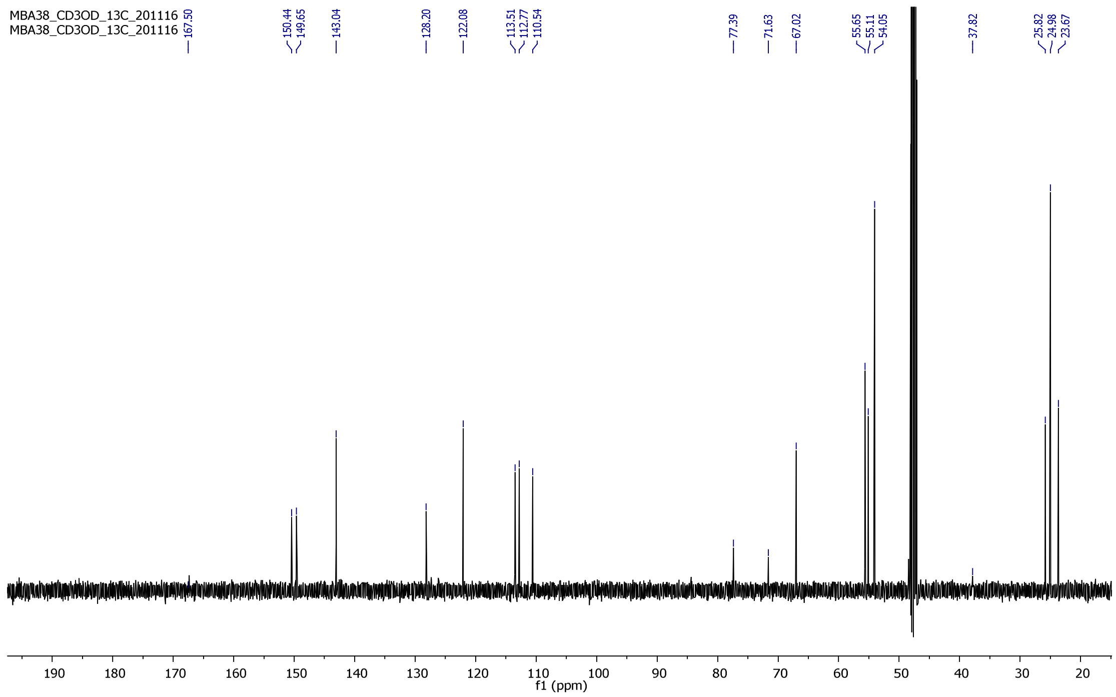

2D COSY NMR

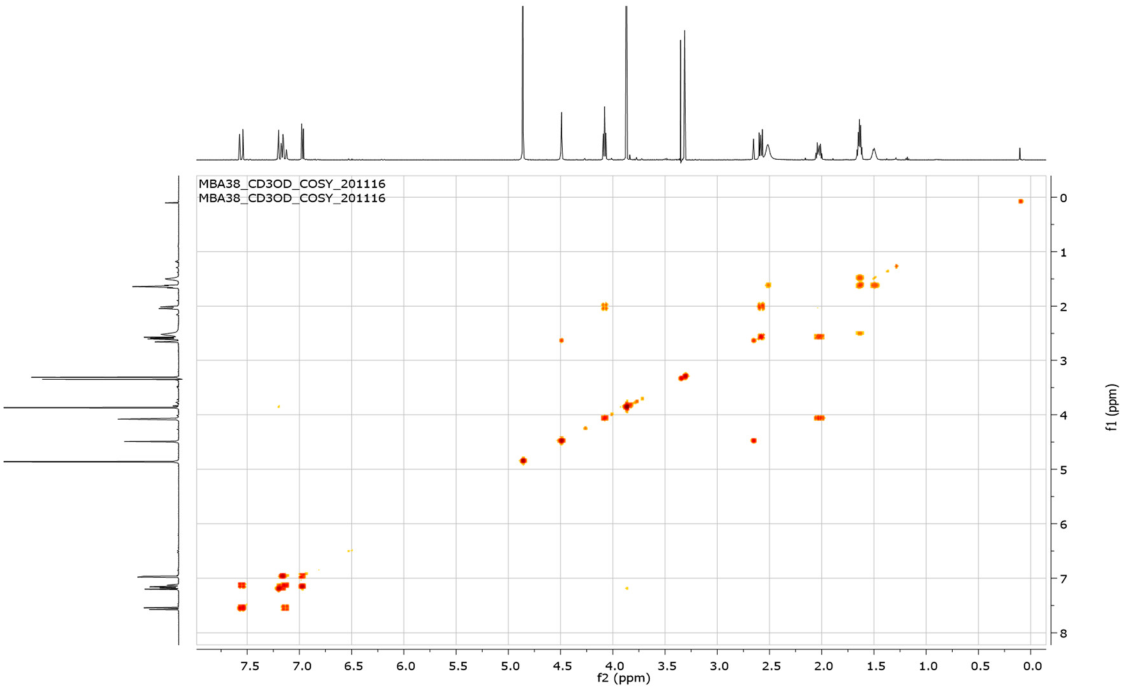

2D HSQC NMR

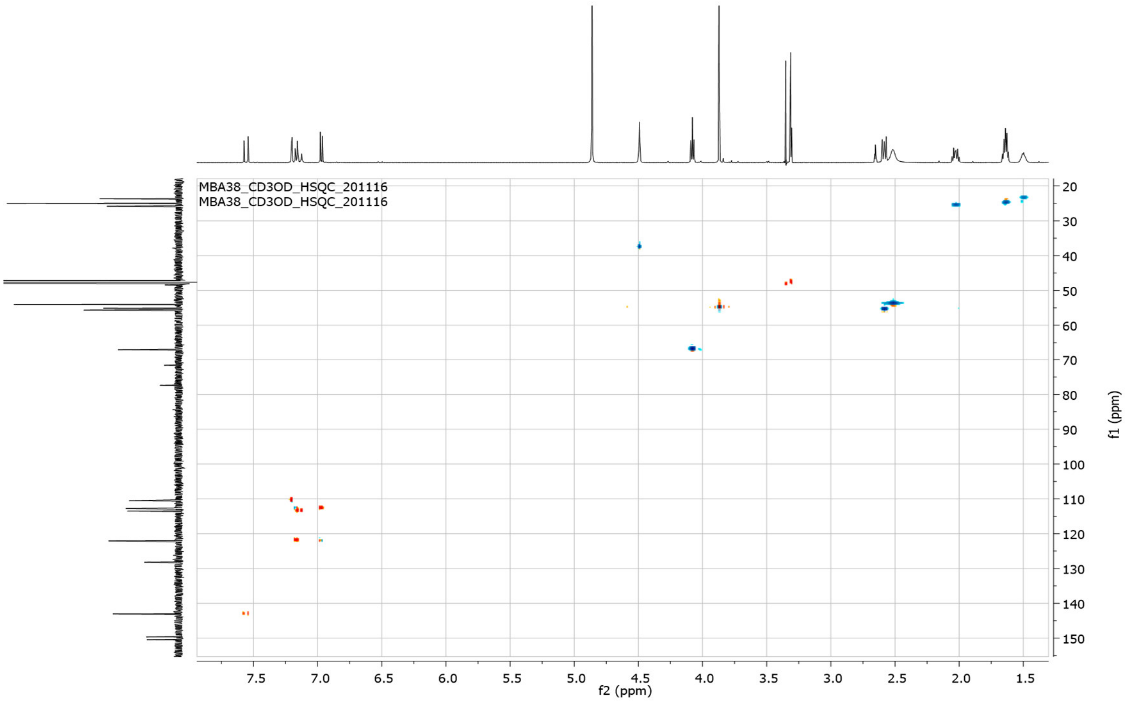

2D HMBC NMR

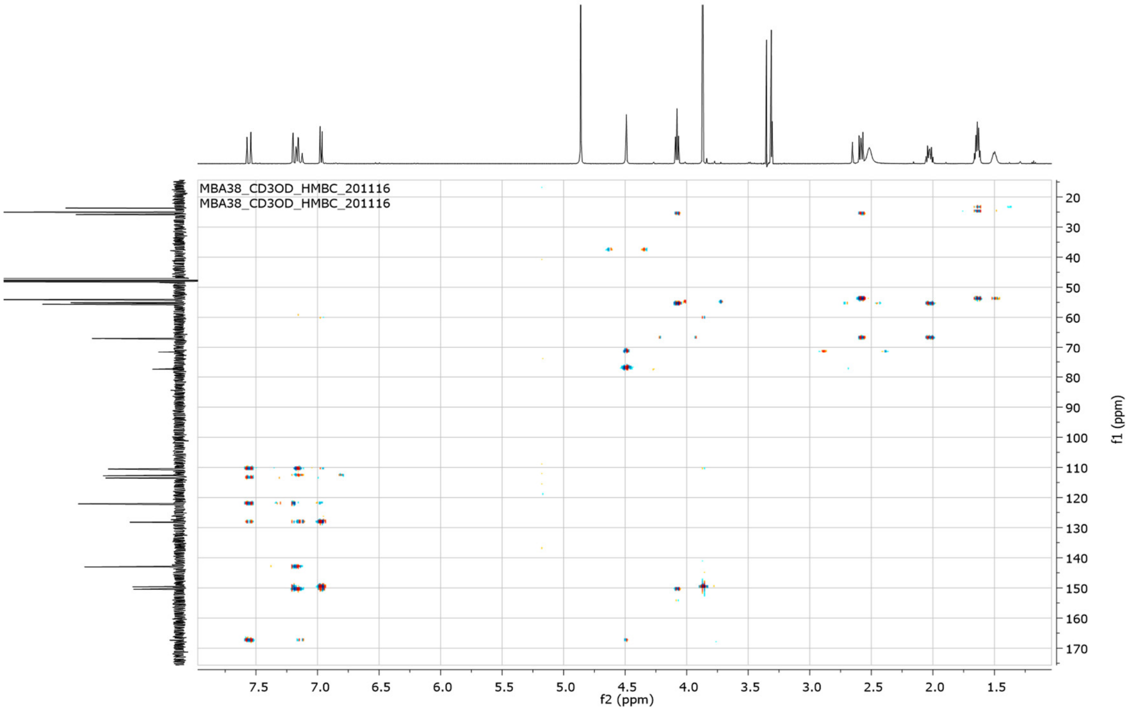

FTIR

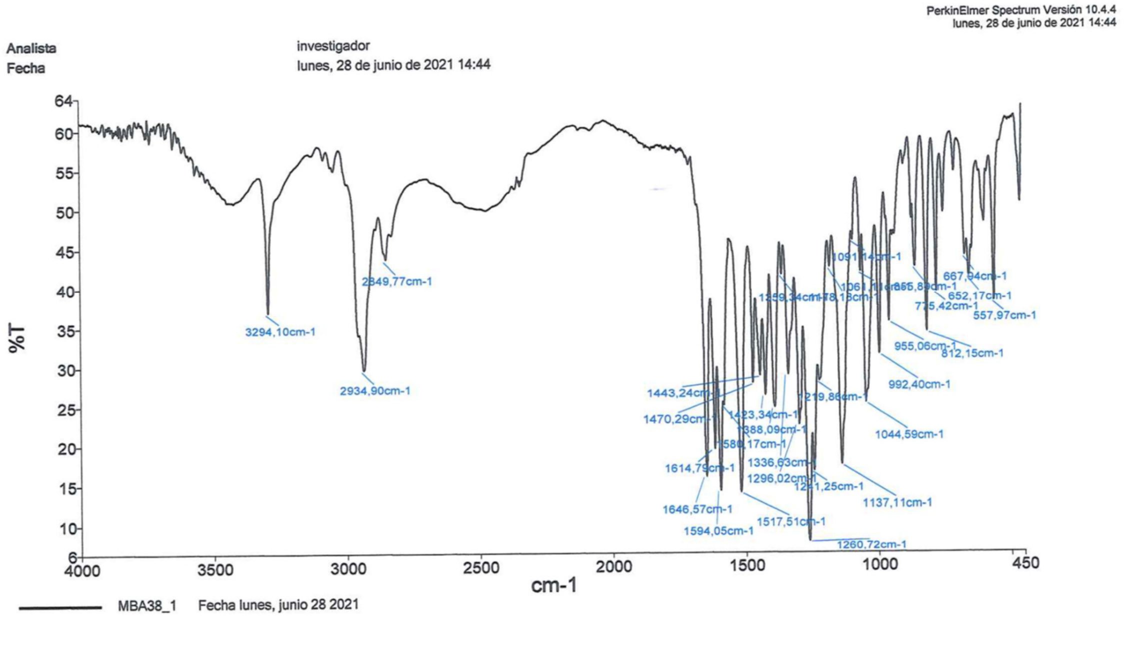

HRMS

Qualitative Compound Report

**Data File**	249_MBA38_01.d	**Sample Name**	MBA38
**Sample Type**	Sample	**Position**	Vial 3
**Instrument Name**	Instrument 1	**User Name**	
**Acq Method**	ESI_ACN_75_pos_new.m	**Acquired Time**	2/19/2021 11:14:26 AM (UTC+01:00)
**IRM Calibration Status**	Success	**DA Method**	Defecto_modificado.m
**Comment**			

**Sample Group**		**Info.**	
**User**	DANIEL DIEZ	**Stream Name**	LC 1
**Acquisition Time (Local)**	2/19/2021 11:14:26 AM (UTC+01:00)	**Acquisition SW Version**	6200 series TOF/6500 series Q-TOF B.08.00 (B8058.3 SP1)
**QTOF Driver Version**	8.00.00	**QTOF Firmware Version**	2.712
**Tune Mass Range Max.**	1700		

Compound Table

Compound Label	RT	Mass	Abund	Formula	Tgt Mass	Diff (ppm)	Hits (DB)
Cpd 1: C21 H28 N2 O4; 0.257	0.257	372.2035	1431751	C21 H28 N2 O4	372.2049	-3.66	1

Compound Label	*m/z*	RT	Algorithm	Mass
Cpd 1: C21 H28 N2 O4; 0.257	373.2093	0.257	Find by Formula	372.2035

MS Zoomed Spectrum

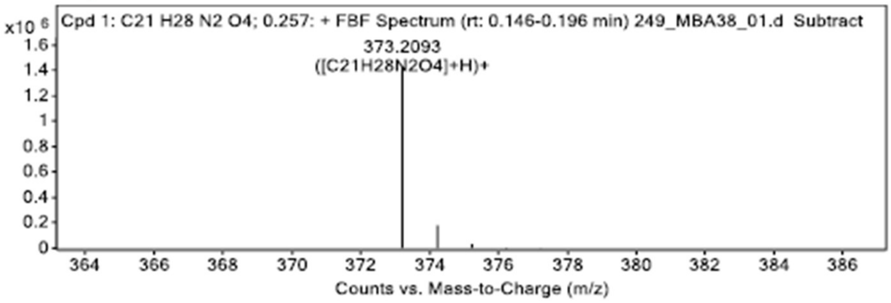

MS Spectrum Peak List

m/z	z	Abund	Formula	Ion
373.2093	1	1431750.88	C21H28N2O4	(M+H)+
374.2238	1	181837.64	C21H28N2O4	(M+H)+
375.2262	1	32809.93	C21H28N2O4	(M+H)+
376.2323	1	4496.41	C21H28N2O4	(M+H)+
377.2283	1	738.6	C21H28N2O4	(M+H)+

MS Zoomed Spectrum

Qualitative Compound Report

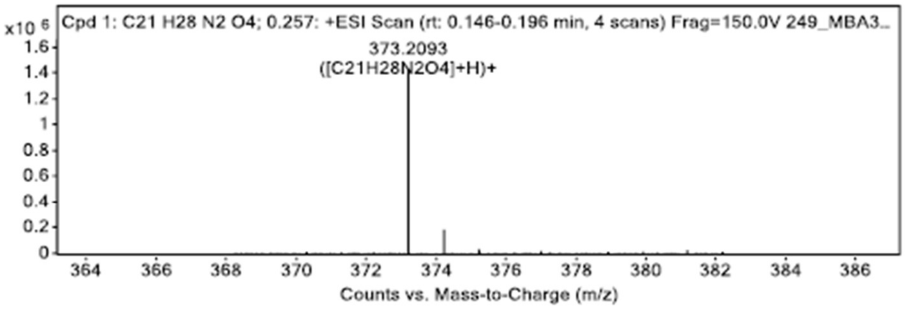

MS Spectrum Peak List

m/z	*Calc m/z*	Diff(ppm)	z	Abund	Formula	Ion
373.2093	373.2122	-7.68	1	1431750.88	C21H28N2O4	(M+H)+
374.2238	374.2154	22.55	1	181837.64	C21H28N2O4	(M+H)+
375.2262	375.2181	21.56	1	32809.93	C21H28N2O4	(M+H)+
376.2323	376.2207	30.71	1	4496.41	C21H28N2O4	(M+H)+
377.2283	377.2233	13.24	1	738.6	C21H28N2O4	(M+H)+

--- End Of Report ---

Compound 6

^{1}H NMR

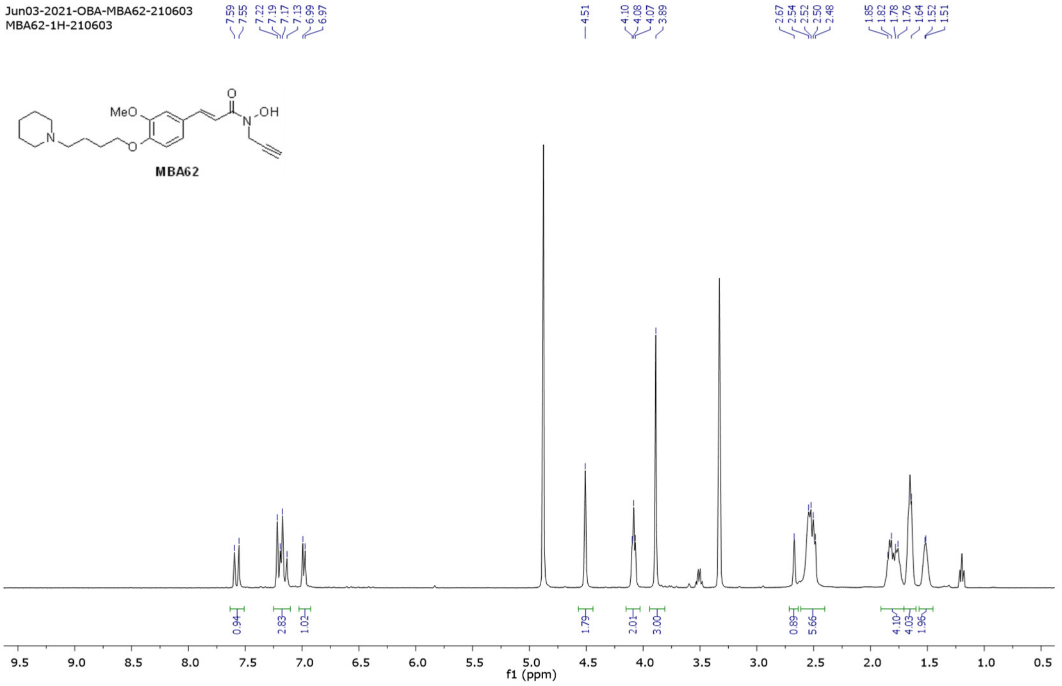

^{13}C NMR

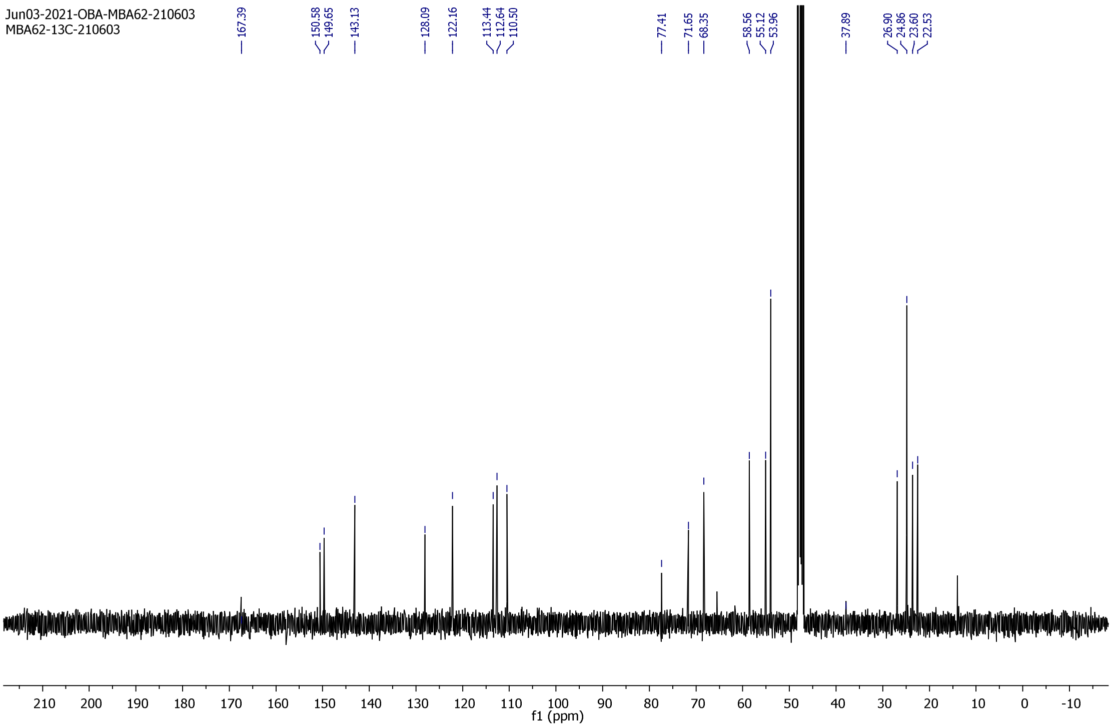

2D COSY NMR

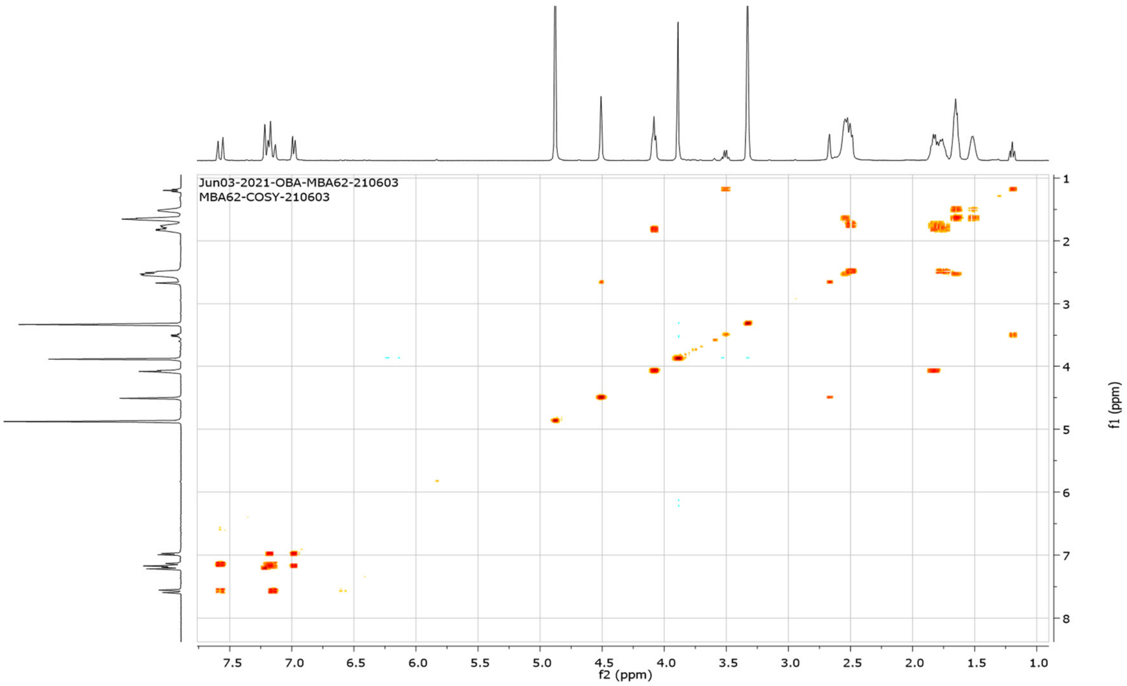

2D HSQC NMR

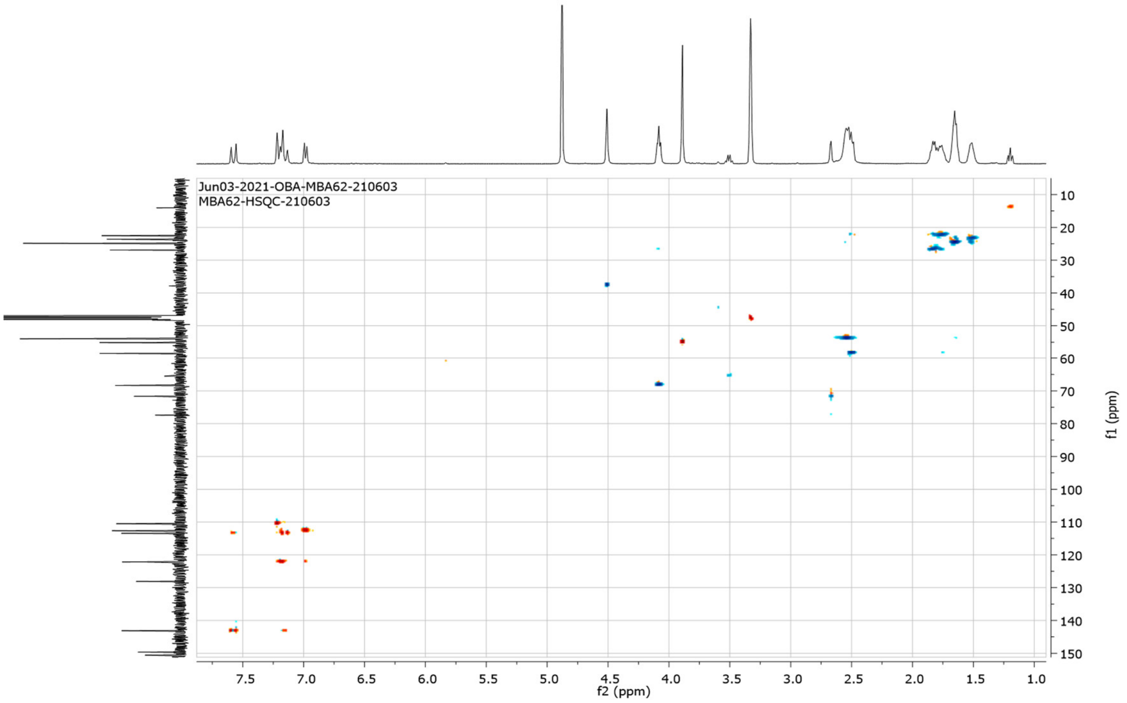

2D HMBC NMR

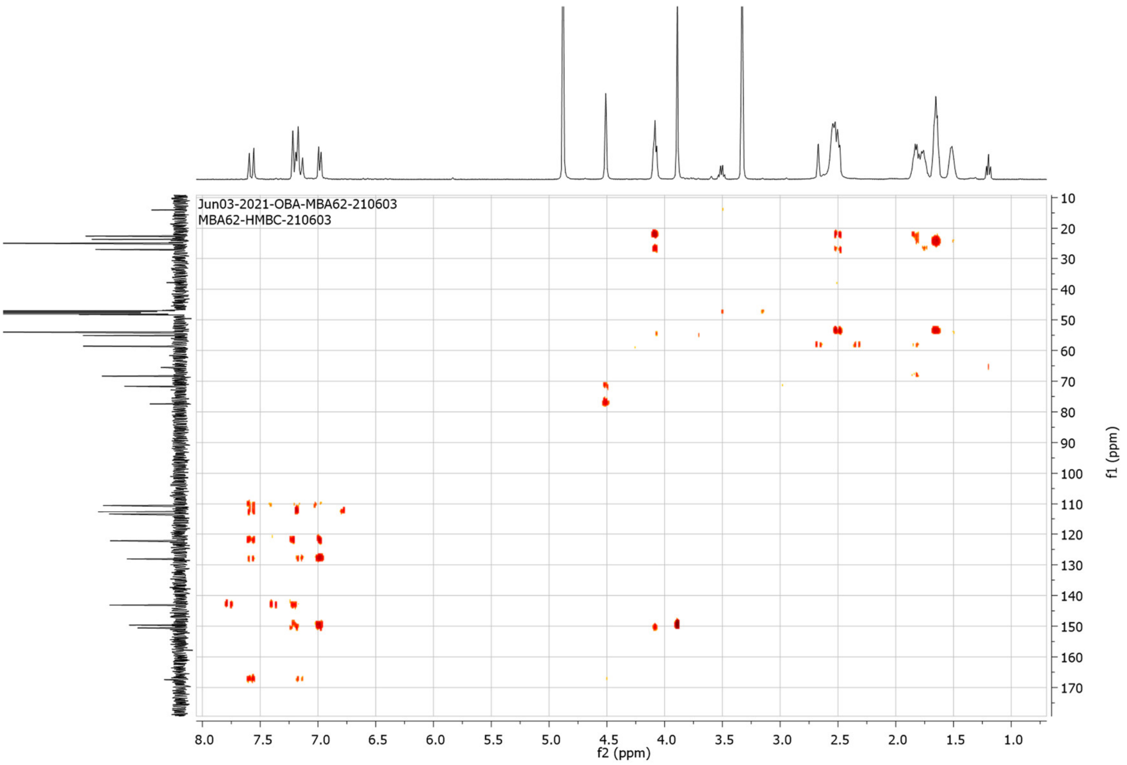

FTIR

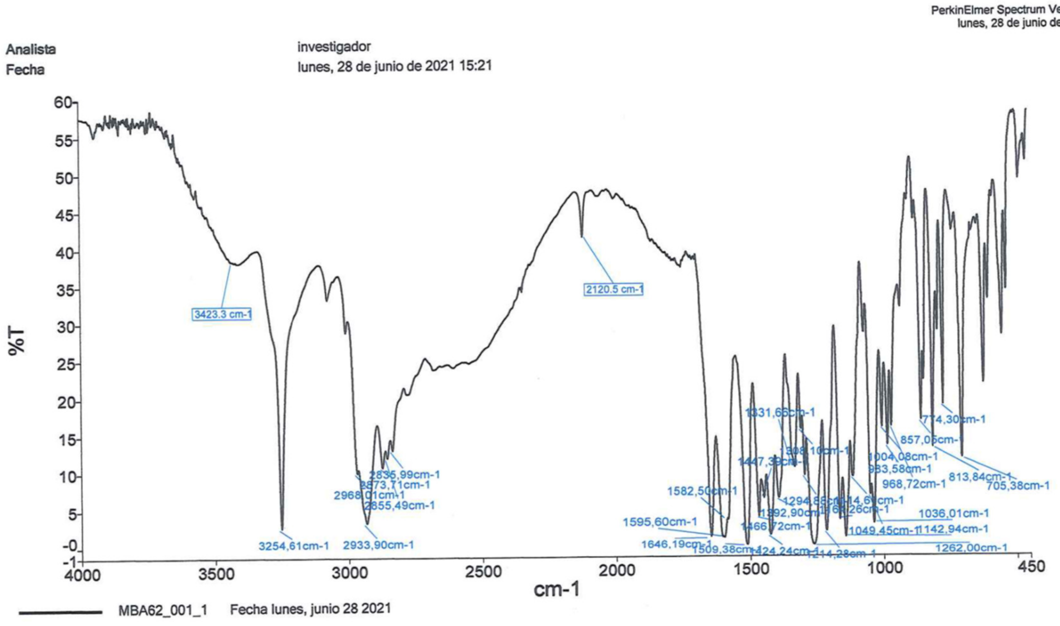

HRMS

Qualitative Compound Report

**Data File**	538_MBA62_01.d	**Sample Name**	MBA62
**Sample Type**	Sample	**Position**	Vial 15
**Instrument Name**	Instrument 1	**User Name**	
**Acq Method**	ESI_ACN_75_pos_new.m	**Acquired Time**	6/1/2021 1:27:43 PM (UTC+01:00)
**IRM Calibration Status**	Success	**DA Method**	Defecto_modificado.m
**Comment**			

**Sample Group**		**Info.**	
**User**	MIREIA TOLEDANO	**Stream Name**	LC 1
**Acquisition Time (Local)**	6/1/2021 1:27:43 PM (UTC+01:00)	**Acquisition SW Version**	6200 series TOF/6500 series Q-TOF B.08.00 (B8058.3 SP1)
**QTOF Driver Version**	8.00.00	**QTOF Firmware Version**	2.712
**Tune Mass Range Max.**	1700		

Compound Table

Compound Label	RT	Mass	Abund	Formula	Tgt Mass	Diff (ppm)	Hits (DB)
Cpd 1: C22 H30 N2 O4; 0.232	0.232	386.2204	9910694	C22 H30 N2 O4	386.2206	-0.5	1

Compound Label	*m/z*	RT	Algorithm	Mass
Cpd 1: C22 H30 N2 O4; 0.232	387.2277	0.232	Find by Formula	386.2204

MS Zoomed Spectrum

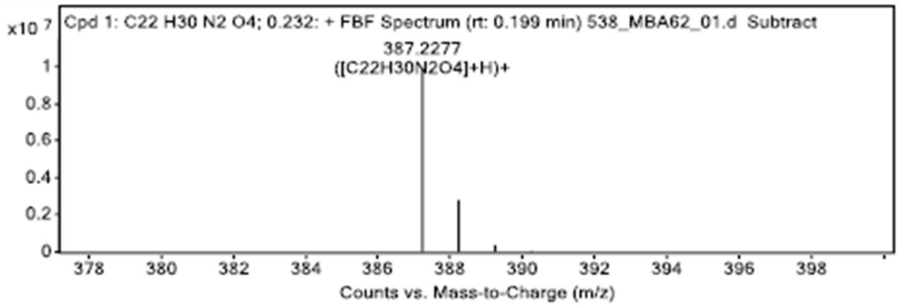

MS Spectrum Peak List

m/z	z	Abund	Formula	Ion
387.2277	1	9910694	C22H30N2O4	(M+H)+
388.2304	1	2801086.25	C22H30N2O4	(M+H)+
389.2357	1	376968.97	C22H30N2O4	(M+H)+
390.2394	1	43281.25	C22H30N2O4	(M+H)+

MS Zoomed Spectrum

Qualitative Compound Report

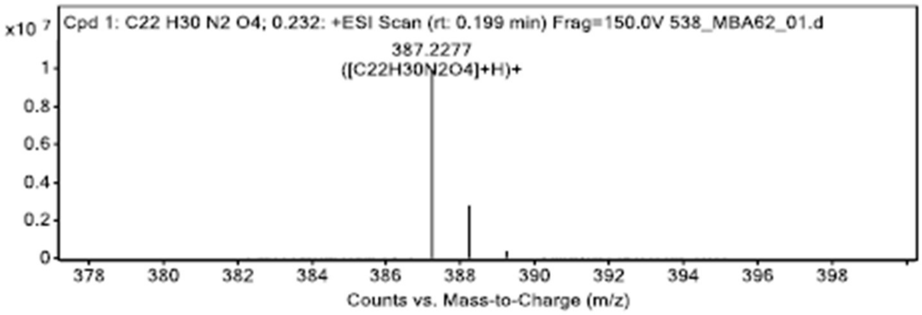

MS Spectrum Peak List

m/z	*Calc m/z*	Diff(ppm)	z	Abund	Formula	Ion
387.2277	387.2278	-0.38	1	9910694	C22H30N2O4	(M+H)+
388.2304	388.2311	-1.78	1	2801086.25	C22H30N2O4	(M+H)+
389.2357	389.2338	4.92	1	376968.97	C22H30N2O4	(M+H)+
390.2394	390.2364	7.45	1	43281.25	C22H30N2O4	(M+H)+

--- End Of Report ---

Compound 15

^{1}H NMR

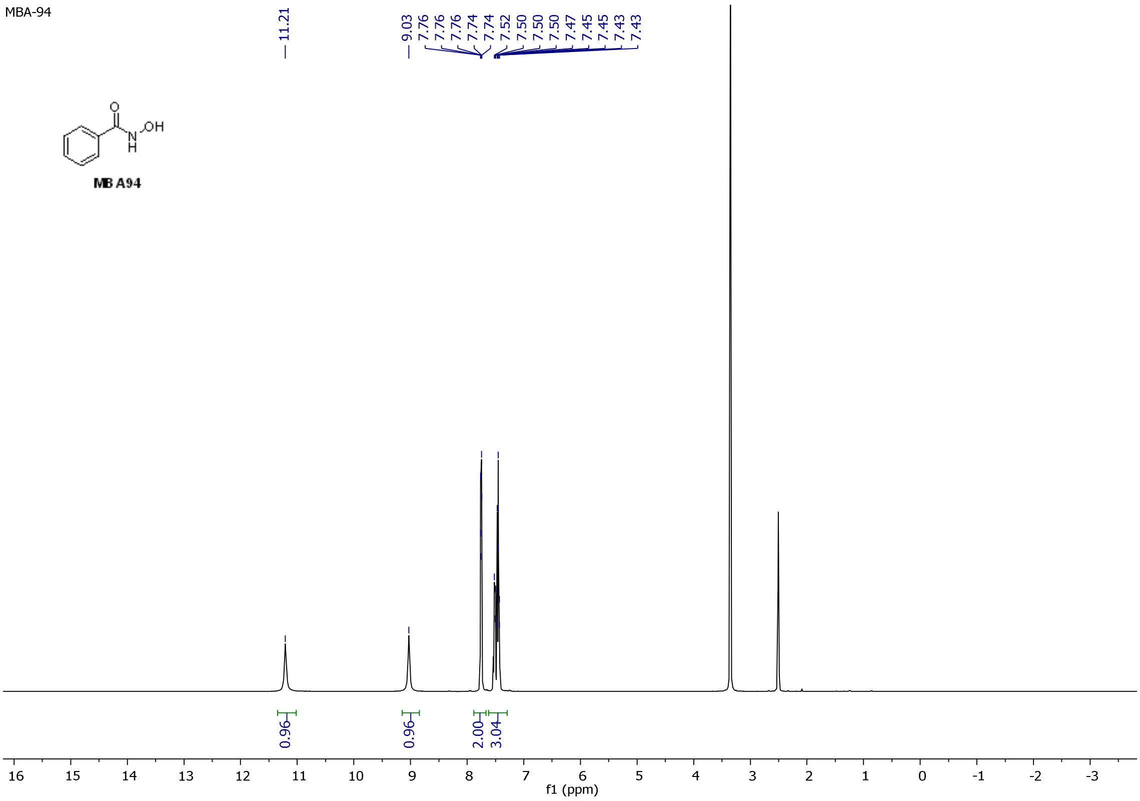

^{13}C NMR

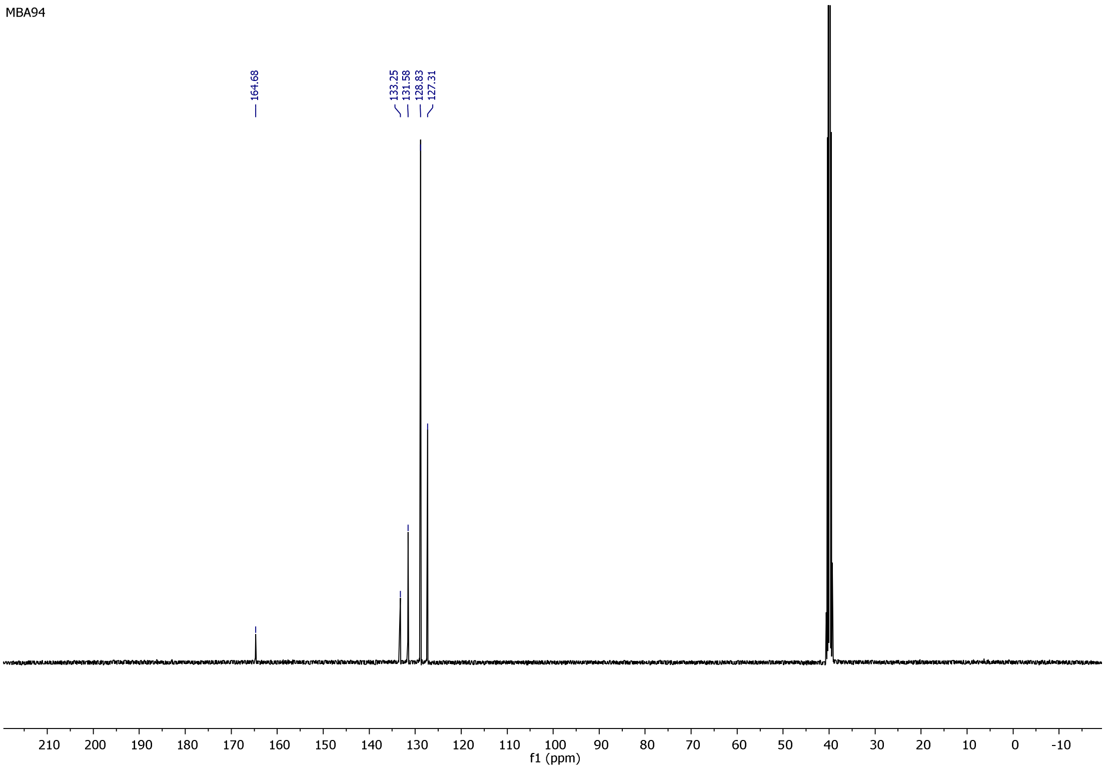

HRMS

Qualitative Compound Report

**Data File**	815_MBA94_02.d	**Sample Name**	
**Sample Type**		**Position**	
**Instrument Name**		**User Name**	
**Acq Method**		**Acquired Time**	
**IRM Calibration Status**	Success	**DA Method**	Defecto_modificado.m
**Comment**			

Status Acquisition is not yet complete.

Compound Table

Compound Label	RT	Mass	Abund	Formula	Tgt Mass	Diff (ppm)	Hits (DB)
Cpd 1: C7 H7 N O2; 0.251	0.251	137.0478	914665	C7 H7 N O2	137.0477	0.59	1

Compound Label	*m/z*	RT	Algorithm	Mass
Cpd 1: C7 H7 N O2; 0.251	138.0548	0.251	Find by Formula	137.0478

MS Zoomed Spectrum

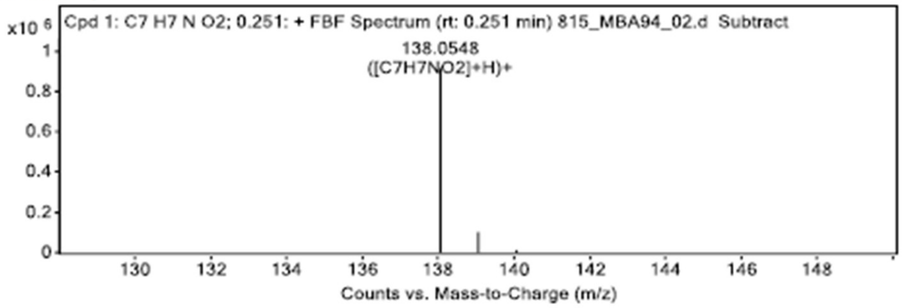

MS Spectrum Peak List

m/z	z	Abund	Formula	Ion
138.0548	1	914664.94	C7H7NO2	(M+H)+
139.0594	1	102169.3	C7H7NO2	(M+H)+
140.0681	1	11388	C7H7NO2	(M+H)+

MS Zoomed Spectrum

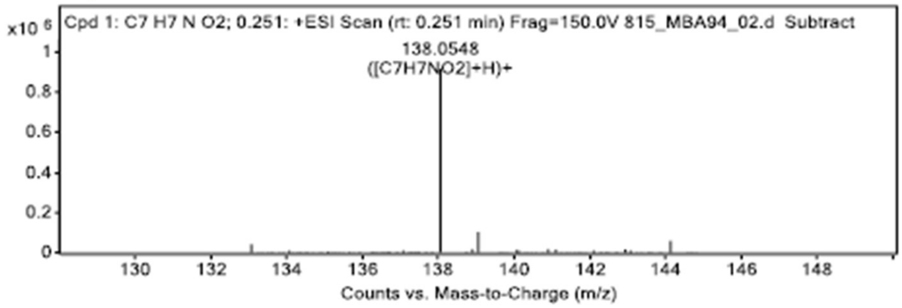

MS Spectrum Peak List

m/z	*Calc m/z*	Diff(ppm)	z	Abund	Formula	Ion
138.0548	138.055	-1.18	1	914664.94	C7H7NO2	(M+H)+
139.0594	139.0581	9.77	1	102169.3	C7H7NO2	(M+H)+

Qualitative Compound Report

140.0681	140.06	57.93	1	11388	C7H7NO2	(M+H)+

--- End Of Report ---

FTIR

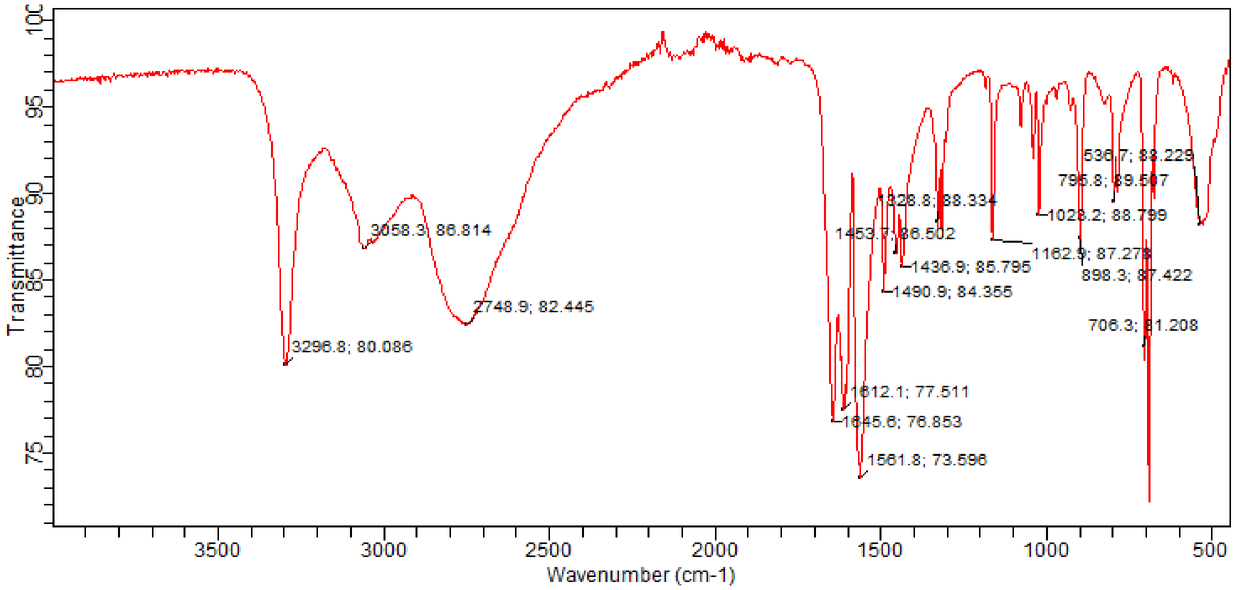

Transmittance
Wavenumber (cm-1)
3296.8; 80.086
3058.3; 86.814
2748.9; 82.445
1645.6; 76.853
1612.1; 77.511
1561.8; 73.596
1490.9; 84.355
1453.7; 86.502
1436.9; 85.795
1328.8; 88.334
1162.9; 87.278
1028.2; 88.799
898.3; 87.422
795.8; 89.507
706.3; 81.208
536.7; 88.229

Compound 16

^{1}H NMR

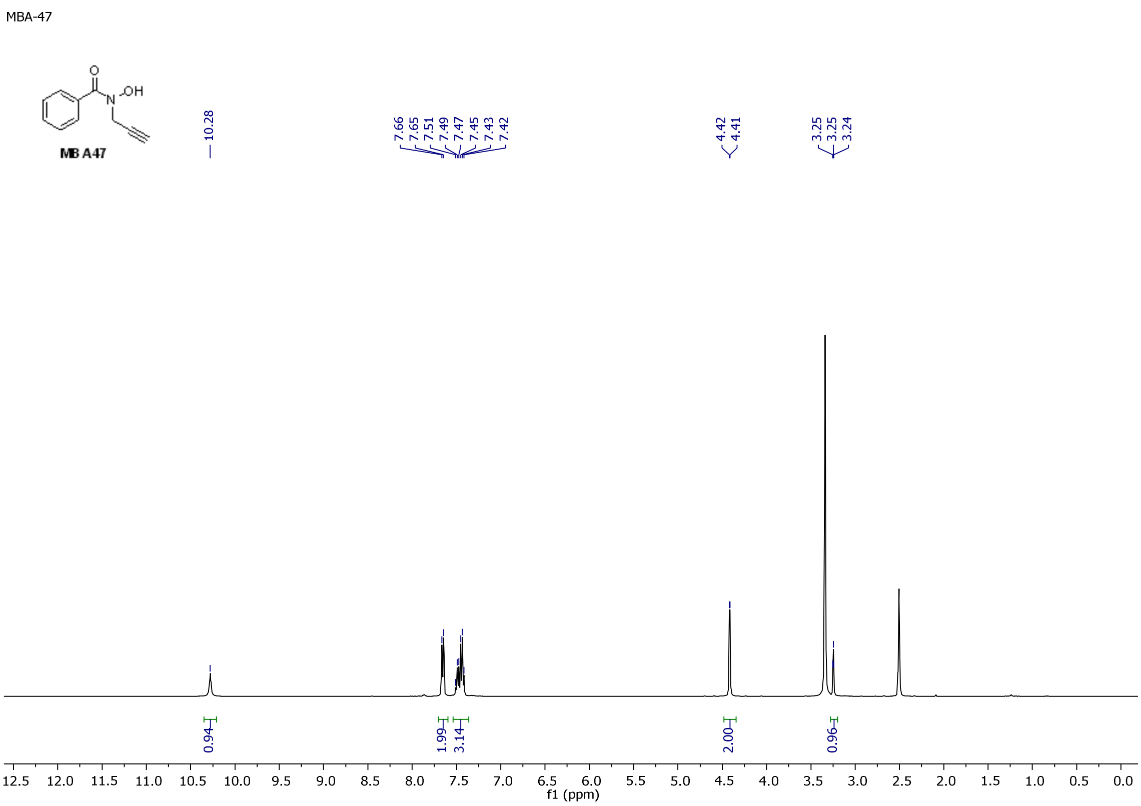

^{13}C NMR

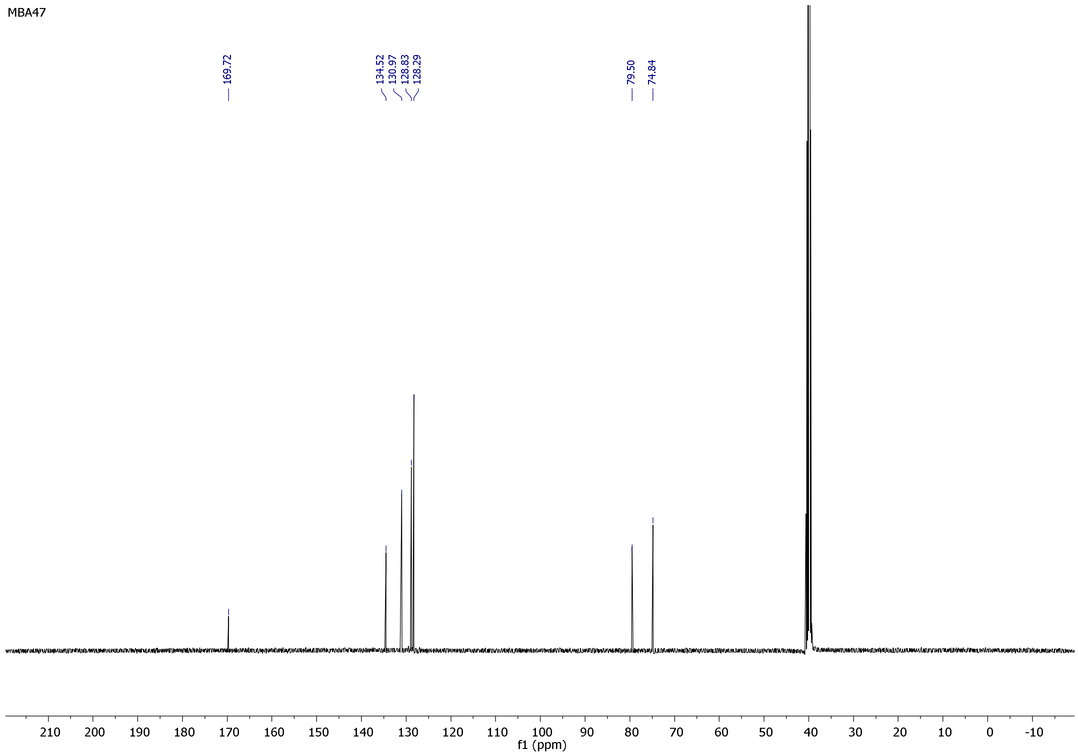

HRMS

Qualitative Compound Report

**Data File**	814_MBA47_01.d	**Sample Name**	MBA47
**Sample Type**	Sample	**Position**	Vial 7
**Instrument Name**	Instrument 1	**User Name**	
**Acq Method**	ESI_ACN_75_pos_new.m	**Acquired Time**	9/10/2021 3:14:00 PM (UTC+01:00)
**IRM Calibration Status**	Success	**DA Method**	Defecto_modificado.m
**Comment**			

**Sample Group**		**Info.**	
**User**	MIREIA TOLEDANO	**Stream Name**	LC 1
**Acquisition Time (Local)**	9/10/2021 3:14:00 PM (UTC+01:00)	**Acquisition SW Version**	6200 series TOF/6500 series Q-TOF B.08.00 (B8058.3 SP1)
**QTOF Driver Version**	8.00.00	**QTOF Firmware Version**	2.712
**Tune Mass Range Max.**	1700		

Compound Table

Compound Label	RT	Mass	Abund	Formula	Tgt Mass	Diff (ppm)	Hits (DB)
Cpd 1: C10 H9 N O2; 0.244	0.244	175.0626	927273	C10 H9 N O2	175.0633	-4.17	1

Compound Label	*m/z*	RT	Algorithm	Mass
Cpd 1: C10 H9 N O2; 0.244	176.0696	0.244	Find by Formula	175.0626

MS Zoomed Spectrum

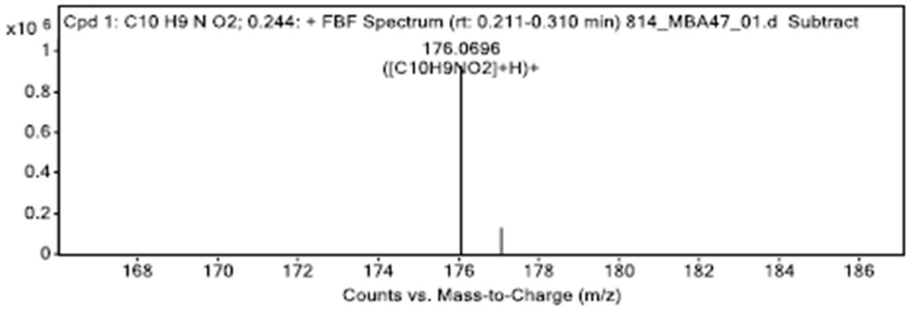

MS Spectrum Peak List

m/z	z	Abund	Formula	Ion
176.0696	1	927273.38	C10H9NO2	(M+H)+
177.0748	1	129039.83	C10H9NO2	(M+H)+

MS Zoomed Spectrum

Qualitative Compound Report

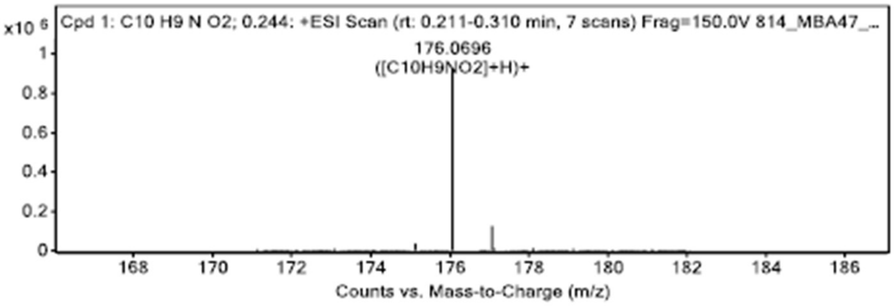

MS Spectrum Peak List

m/z	*Calc m/z*	Diff(ppm)	z	Abund	Formula	Ion
176.0696	176.0706	-5.53	1	927273.38	C10H9NO2	(M+H)+
177.0748	177.0738	5.75	1	129039.83	C10H9NO2	(M+H)+

--- End Of Report ---

FTIR

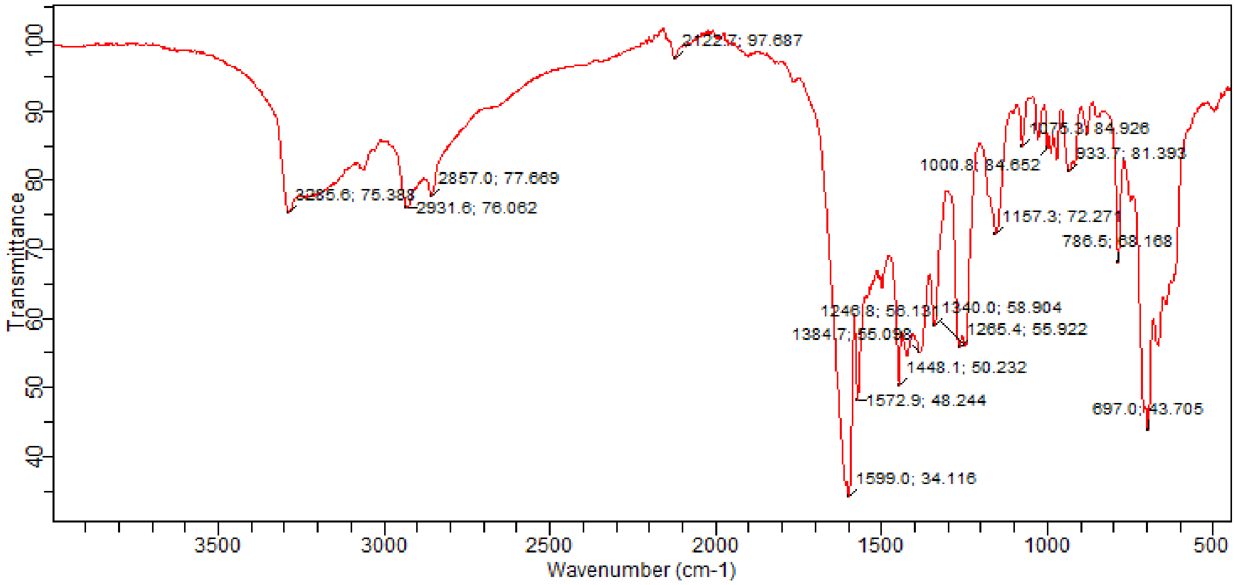

Transmittance
Wavenumber (cm-1)
3285.6; 75.388
2931.6; 76.062
2857.0; 77.669
2122.7; 97.687
1599.0; 34.116
1572.9; 48.244
1448.1; 50.232
1384.7; 55.098
1246.8; 56.131
1340.0; 58.904
1265.4; 55.922
1157.3; 72.271
1000.8; 84.652
1075.3; 84.926
933.7; 81.393
786.5; 68.168
697.0; 43.705
